# Targeting the tumor microenvironment in B-cell lymphoma: challenges and opportunities

**DOI:** 10.1186/s13045-021-01134-x

**Published:** 2021-08-17

**Authors:** Yingyue Liu, Xiangxiang Zhou, Xin Wang

**Affiliations:** 1 Department of Hematology, Shandong Provincial Hospital, Cheeloo College of Medicine, Shandong University, No. 324, Jingwu Road, Jinan, 250021 Shandong China; 2 Department of Hematology, Shandong Provincial Hospital Affiliated to Shandong First Medical University, Jinan, 250021 Shandong China; 3 School of Medicine, Shandong University, Jinan, 250012 Shandong China; 4 Shandong Provincial Engineering Research Center of Lymphoma, Jinan, 250021 Shandong China; 5 Branch of National Clinical Research Center for Hematologic Diseases, Jinan, 250021 Shandong China; 6 National Clinical Research Center for Hematologic Diseases, The First Affiliated Hospital of Soochow University, Suzhou, 251006 China

**Keywords:** Tumor microenvironment, B-cell lymphoma, Immunosuppression, Targeted therapy

## Abstract

B-cell lymphoma is a group of hematological malignancies with high clinical and biological heterogeneity. The pathogenesis of B-cell lymphoma involves a complex interaction between tumor cells and the tumor microenvironment (TME), which is composed of stromal cells and extracellular matrix. Although the roles of the TME have not been fully elucidated, accumulating evidence implies that TME is closely relevant to the origination, invasion and metastasis of B-cell lymphoma. Explorations of the TME provide distinctive insights for cancer therapy. Here, we epitomize the recent advances of TME in B-cell lymphoma and discuss its function in tumor progression and immune escape. In addition, the potential clinical value of targeting TME in B-cell lymphoma is highlighted, which is expected to pave the way for novel therapeutic strategies.

## Introduction

Lymphomas mainly comprise Hodgkin lymphoma (HL) and non-Hodgkin lymphoma (NHL), representing a heterogeneous group of lymphoproliferative diseases. B-cell lymphomas account for almost 95% of all lymphoma cases [1], among which diffuse large B-cell lymphoma (DLBCL) is the most common subtype, accounting for approximately 30% of all NHL cases [2]. Patients with B-cell lymphomas are usually characterized by lymphadenopathy, extranodal disease or both and present the potential for multiple organ involvement. Therefore, early diagnosis and therapy are essential. With the development of molecular diagnosis techniques, efforts have been made to better classify B-cell lymphoma. However, due to the heterogeneity of this disease, only a few strategies are applied to routine diagnosis and prognosis prediction.

The tumor microenvironment (TME) is a complex network that comprises cellular and noncellular components, forming a physical barrier around tumor cells [3]. Accumulating studies have suggested that the TME components play important roles in the initiation and maintenance of carcinogenesis instead of being bystanders [4]. TME is instrumental in a variety of biological processes, including pathogenesis, progression, metastasis and drug resistance, through sustained proliferation and immune escape [5]. Given the limited efficacy of standard therapies in several patients, TME-based therapies have been explored as new treatment strategies to achieve a more immunogenic environment and better drug delivery, ultimately increasing the response rates of patients. Recent studies suggest that the composition of TME is essential for the pathogenesis of lymphoma. Moreover, the TME also provides new strategies for targeted therapies and tumor prognosis prediction. In summary, this review may offer novel therapeutic strategies for B-cell lymphoma through describing the essential elements of the TME, TME–targeted therapy and clinical applications, and novel technologies applied in TME detection.

### Composition of TME in B-cell lymphoma

**Fig. 1:**
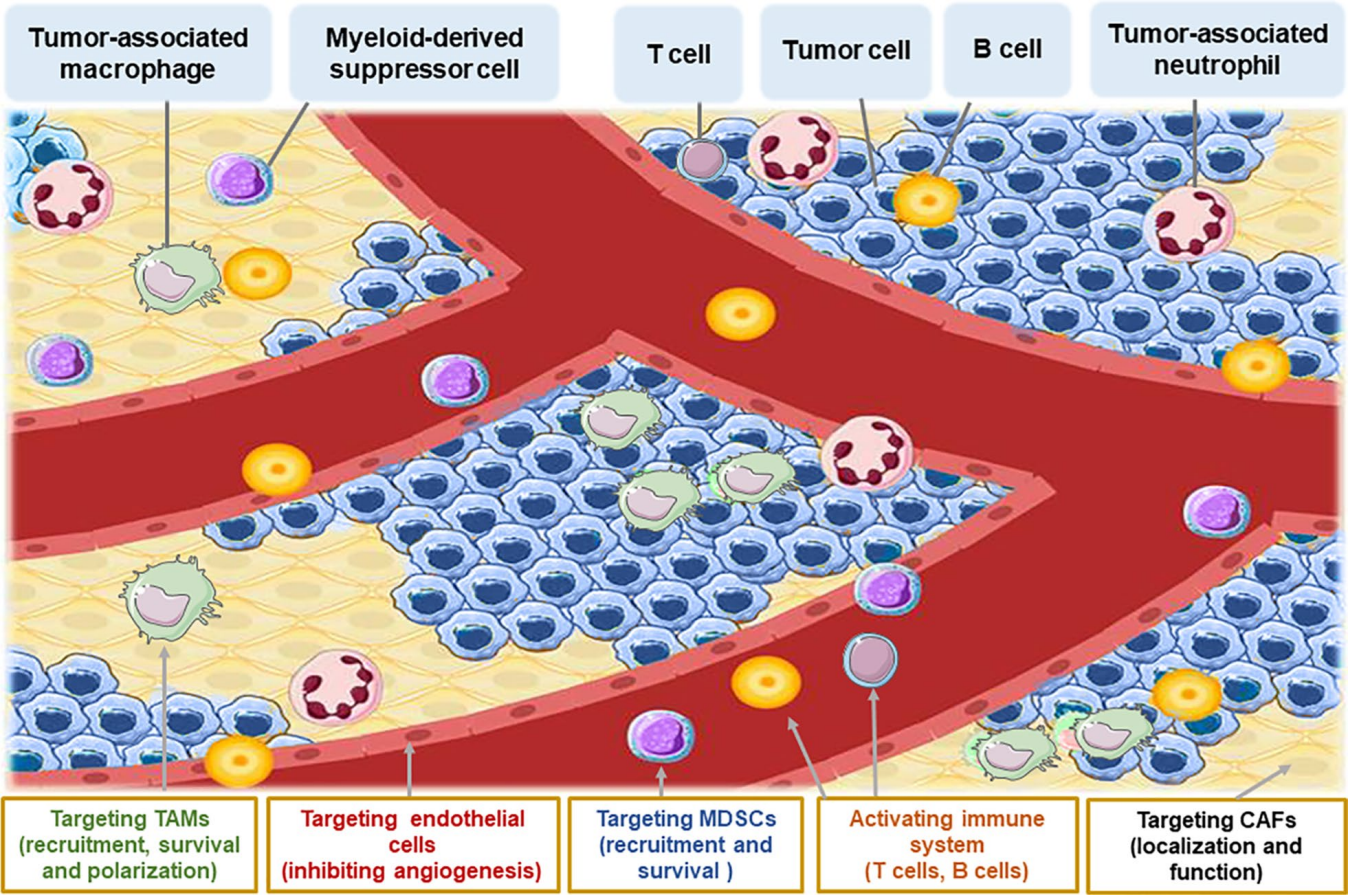
Major constituents of the TME and targeted therapies. The figure depicts the typical microenvironment of B-cell lymphoma. Tumor microenvironment refers to the internal environment in which tumor cells produce and live, major cellular and noncellular components. It includes not only the tumor cells but also the immune and inflammatory cells, fibroblasts and other cells around them. It also comprises the intercellular substance, micro-vessels and biological molecules infiltrated in the nearby area. Current strategies targeting TME components are also highlighted

The TME can be divided into two parts: the immune microenvironment that contains immune cells and the nonimmune microenvironment dominated by fibroblasts [6]. The immune microenvironment consists of T and B lymphocytes, tumor-associated macrophages (TAMs), myeloid-derived suppressor cells (MDSCs), tumor-associated neutrophils (TANs), natural killer (NK) cells, dendritic cells (DCs) and others. These cells mediate the immunosuppressive microenvironment and escape immunity. The nonimmune microenvironment mainly consists of stromal cells, including cancer-associated fibroblasts (CAFs), extracellular matrix (ECM), pericytes, mesenchymal stromal cells and other secreted molecules, including growth factors, cytokines, chemokines and extracellular vesicles (Fig. 1) [7]. These TME cells express different biomarkers and play a variety of roles in the tumorigenesis and prognosis of B-cell lymphoma (Table 1).

**Table 1:** Markers and functions of cells in TME

Cell type	Common markers		Major role in tumors	Effects in B-cell lymphoma	References
TAMs	M1	CD68^+^ CD80^+^ CD86^+^	Anti-tumorigenic	DLBCL: M2 TAMs correlate with poor prognosis HL: CD68^+^ TAMs correlate with poor prognosis FL: High CD68^+^ TAMs correlate with longer survival rates after R-CHOP	[8–10]
	M2	CD163^+^ CD204^+^ CD206^+^	Pro-tumorigenic		
MDSCs	PMN-MDSCs	CD11b^+^ CD14^−^ CD15^+^ CD66^+^	Pro-tumorigenic	DLBCL: M-MDSCs correlate with poor prognosis HL: PMN-MDSCs correlate with poor prognosis FL: Unknown	[11–13]
	M-MDSCs	Lin^−^ CD11b^+^ CD14^+^ HLA-DR^low^			
TANs	N1	CD16^+^ CD66b^+^ CD170^low^	Anti-tumorigenic	DLBCL: TANs correlate with poor prognosis HL: High neutrophil‐lymphocyte ratio correlates with poor prognosis in NS-cHL FL: Unknown	[14–16]
	N2	CD66b^+^ CD11b^+^ CD170^high^	Pro-tumorigenic		
NK cells	bone marrow NK cells	CD56^bright^ CD16^−^	Anti-tumorigenic	DLBCL: Low NK cells correlate with shorter PFS HL: Unknown FL: Low peripheral blood NK cells count correlate with shorter PFS and OS	[17, 18]
	mature NK cells	CD56^dim^ CD16^+^			
CAFs	α-SMA^+^ S100A4^+^ FAP^+^ CD10^+^		Pro-tumorigenic	DLBCL: CAFs associate with favorable prognosis HL: CAFs protect Hodgkin and Reed–Sternberg cells from Brentuximab-Vedotin induced injury in NS-cHL FL: CAFs correlate with poor prognosis	[19–22]
DCs	pDCs	CD123^+^ CD303^+^ CD304^+^	Pro-tumorigenic	DLBCL: DCs correlate with longer survival HL: Unknown FL: Follicular DCs correlate with poor prognosis	[23–25]
	mDCs	CD1c^+^ CD141^+^			

TME, tumor microenvironment; TAMs, tumor-associated macrophages; MDSCs, myeloid-derived suppressor cells; PMN-MDSCs, polymorphonuclear myeloid-derived suppressor cells; M-MDSCs, monocytic myeloid-derived suppressor cells; TANs, tumor-associated neutrophils; NK cells, natural killer cells; CAFs, cancer-associated fibroblasts; DCs, dendritic cells; pDCs, plasmacytoid dendritic cells; mDCs, myeloid dendritic cells; DLBCL, diffuse large B-cell lymphoma; HL, Hodgkin lymphoma; FL, follicular lymphoma; cHL, classical Hodgkin lymphoma; NS-cHL, nodular sclerosis classical Hodgkin lymphoma

#### TAMs

As the most intensive immunosuppressive cell populations in the TME, TAMs were responsible for the inhibition of recruitment and activation of T cells via secreting cytokines, chemokines and other factors, thereby promoting immunosuppression [26]. TAMs are usually classified into M1 and M2 phenotypes. The classification and identification of TAMs are related to their immunomodulatory functions [27]. M1-like macrophages are considered to be cytotoxic and participate in the antitumor process. However, during the development of tumors, TAMs usually transform into the M2 phenotype [28]. M2-like macrophages produce Th2 cytokines, such as interleukin (IL)-4, IL-10, IL-13, matrix metalloproteinases (MMPs) and transforming growth factor-β (TGF-β), which participate in multiple signaling pathways. TAMs contribute to tumor progression by modulating angiogenesis, immunosuppression and chronic inflammation via complex interaction between signaling pathways [29].

#### MDSCs

MDSCs originate from immature myeloid cells and are divided into two major subsets: polymorphonuclear (PMN)-MDSCs and monocytic (M)-MDSCs. PMN-MDSCs are more similar to neutrophils in morphology and phenotype, whereas the resemblance between M-MDSCs and monocytes is remarkable [30]. TME-derived MDSCs play an intricate role in immunosuppression. Apart from promoting the expansion of regulatory T cells (Tregs), MDSCs also assist in the secretion and activation of inhibitory molecules, such as arginase1 (Arg1), inducible nitric oxide synthase (iNOS), reactive oxygen species (ROS) and prostaglandin E2 (PGE2), thereby suppressing the function of effector T cells [31]. It was recently demonstrated that various characteristics of the TME could stimulate tumor cells to secrete exosomes. Tumor-derived exosomes accelerate the activation and expansion of MDSCs by transporting functional substances, such as microRNAs (miRNAs), IL, TGF-β and PGE2 [31, 32].

#### TANs

TANs, the most abundant circulating leukocytes, could be polarized to the N1 and N2 phenotypes. Owing to the functional plasticity, the neutrophils exert either antitumor or protumor effects. In the early stage of tumorigenesis, TANs are mainly the N1 phenotype and exert antitumor function by activating IL-18 and secreting interferon (IFN)-β. However, TANs usually transform into the N2 phenotype during tumor progression. TANs of the N2-like phenotype promote tumor progression by regulating proliferation, angiogenesis and metastasis of cancer cells [14]. They also inhibit T cell activation by expressing myeloperoxidase, Arg1, ROS and nitric oxide [30, 33]. The specific mechanism of TAN functional type transition in the TME is still unclear, but several studies have demonstrated that TGF-β could polarize neutrophils into the N2 phenotype [34].

#### Tumor-infiltrating NK cells

NK cells, innate cytotoxic lymphocytes of the immune system, contribute to the prevention of viral infection and tumor growth. NK cells contain two subtypes: CD56^dim^ CD16^+^ NK cells and CD56^bright^ CD16^−^ NK cells. The CD56^dim^ subtype can kill other cells and accounts for the majority of NK cells [17]. The function of NK cells is regulated by the dynamic balance between stimulatory and suppressive cell surface receptors [35, 36]. Inhibitory receptors on NK cells could recognize major histocompatibility complex class I (MHC-I) molecules. Therefore, the decreased expression of MHC-I will lead to the activation of NK cells [36]. Natural killer group 2D (NKG2D), an activating receptor, mediates antitumor and antiviral responses through recognizing ligands that are ubiquitously expressed by virus-infected cells and in the TME, such as MICA, MICB or ULBP/RAET1 [35]. Regulation of the above dynamic balance provides an innovative perspective for NK cell-mediated therapy.

#### CAFs

CAFs are a prominent component of TME with significant heterogeneity and plasticity. In various types of cancer, the origins, phenotypes and functions of CAFs are different. However, it is difficult to define CAFs due to the lack of specific markers [37]. CAFs contribute to cancer progression through modulating a variety of biological processes, including angiogenesis, matrix formation and release of growth factors, cytokines and exosomes [38]. In addition, the secretome, matrisome, surfaceome and metabolome of CAFs could also fuel immune evasion, respectively [39]. In contrast, some subtypes of CAFs, such as Slit2^+^ and CD146^+^ CAFs, exert tumor-suppressive effects and even increase sensitivity of chemotherapy [40]. Although there is a growing interest in developing therapeutic strategies for CAFs, several challenges, including the extent of CAFs heterogeneity, the roles of distinct CAFs subtypes and how to selectively target these subtypes, are not fully understood.

#### ECM

ECM represents a protein network surrounding cells, including collagens, proteoglycans, laminin and fibronectin [41]. CAFs are the main source of ECM synthesis and modification [42]. ECM is crucial for tissue homeostasis and normal organ development. Aberrant remodeling of the ECM mediated by collagen deposition or degradation could promote tumor progression. Mechanistically, the remodeled ECM performs various biological functions, including enhancing cell proliferation, increasing cell death resistance and inducing angiogenesis [43, 44].

#### Other components of TME

Despite the importance of the interactions between the above cells and tumor progression, it is notable that other components of the TME could also influence the fate of tumors. Reprogrammed monocytes could accelerate tumor growth by promoting angiogenesis and remodeling the ECM. Different monocyte subsets can also differentiate into TAMs or DCs, which indirectly participate in tumor progression [45]. Immunosuppressive tumor-infiltrating DCs suppress the antitumor immunity of T cells [46]. These results indicate that the TME is an essential intrinsic portion for the regulation of tumor occurrence, development, invasion and metastasis. Thus, understanding the components of the TME involved in tumorigenesis will contribute to developing novel therapeutic strategies.

### Targeting the TME in B-cell lymphoma

#### Targeting components of the TME

**Fig. 2:**
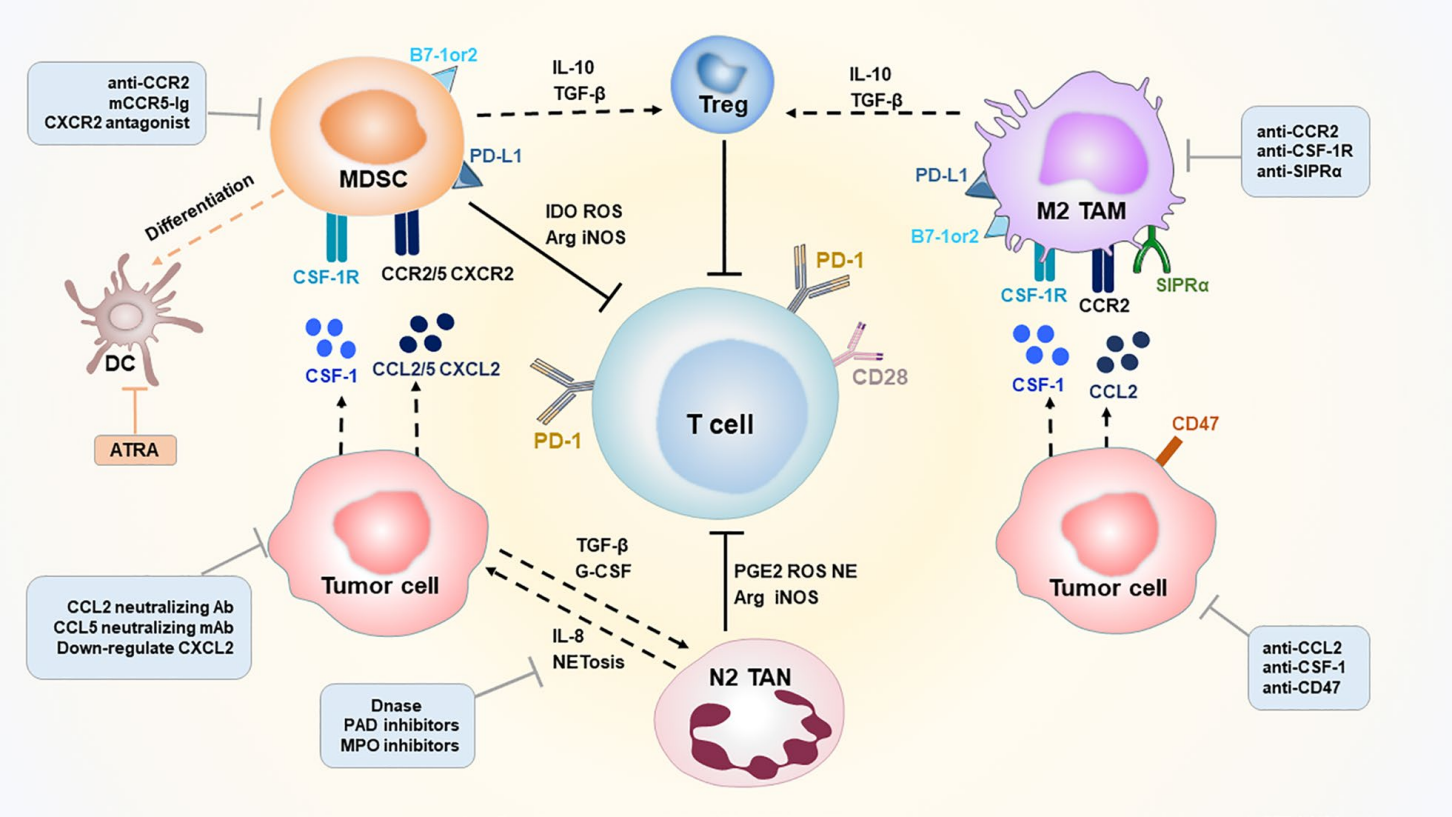
TME targeting strategies to treat B-cell lymphoma. MDSC, TAM (M2), TAN (N2) and Treg inhibit the process of the antitumor immune response through several inhibition pathways and establish an immunosuppressive TME

As mentioned above, the cellular and noncellular components of the TME are involved in tumor progression and the immune response, which provides novel insights for targeted therapies (Fig. 2). Therapeutic strategies are mainly divided into three categories, including depleting existing cells, preventing them from being recruited to tumor sites and reprogramming them into antitumor subtypes [47]. Several promising agents targeting the TME in B-cell lymphoma are summarized in Table 2.

**Table 2:** Clinical trials of agents based on TME cells for B-cell lymphoma treatment

TME cells	Target	Agent	Indication	Phase	Identifier
TAMs	SIRPα	TTI-622	R/R lymphoma R/R multiple myeloma Newly diagnosed acute myeloid leukemia	I	NCT03530683
		GS-0189	R/R NHL	I	NCT04502706
	CD47	Hu5F9-G4	R/R NHL	I	NCT02953509
MDSCs	PI3Kδ/γ	Tenalisib (P65300)	iNHL	II	NCT03711578
TANs	SIRPα	IBI188	Advanced lymphoma	I	NCT03717103
	CD47/CD19	TG-1801	B-cell lymphoma	I	NCT03804996
NK cells		iC9/CAR.19/IL15-transduced CB-NK Cells	B lymphoid malignancies NHL CLL ALL	I/II	NCT03056339
		CD19-targeted high-affinity NK	DLBCL	I	NCT04052061
		AFM13	R/R HL	I	NCT01221571
				II	NCT02321592
	IL-15	ALT-803	R/R iNHL	I/II	NCT02384954
CAFs	FGFR	JNJ-42756493	Advanced or refractory lymphoma	I	NCT01962532

#### Targeting TAMs

Several therapeutic strategies targeting TAMs in B-cell lymphoma are currently being investigated. The colony-stimulating factor-1 (CSF-1)/CSF-1 receptor (CSF-1R) signaling pathway is essential for the recruitment, polarization and functional regulation of TAMs [48]. In mantle cell lymphoma (MCL), the secretion of CSF-1 polarizes monocytes into specific CD163^+^ M2-like TAMs (MϕMCLs), which promotes the proliferation of lymphoma cells. It has been demonstrated that targeting CSF-1R could abrogate MϕMCL-dependent MCL survival [49]. TAMs, also known as nurse-like cells (NLCs), are correlated with the tumorigenesis of chronic lymphocytic leukemia (CLL). Pacritinib, a JAK2/FLT3 inhibitor, was proved to prevent CLL progression by depleting NLCs [50]. Recent studies have verified that CSF-1/CSF-1R blockade improves the efficacy of diverse immunotherapy modalities, such as programmed cell death 1 (PD-1) or cytotoxic T-lymphocyte-associated antigen 4 (CTLA-4) antagonists [51].

TAMs are highly dependent on the CCL2-CCR2 signaling to mobilize from the bone marrow to the site of inflammation in the TME. CCR2 inhibitors induce the accumulation of monocytes in bone marrow, resulting in reduced numbers of TAMs [51]. Yao et al. reported that CREBBP/EP300 mutations could regulate the FBXW7-NOTCH-CCL2/CSF1, polarizing TAMs to the M2 phenotype and promoting cell proliferation in DLBCL [52].

In addition, the combination of CD47 on the surface of tumor cells and SIRPα on TAMs could induce immune escape. Targeting the CD47-SIRPα axis has shown promising results in hematological malignancies [53]. It was recently demonstrated that the therapeutic effect of CD47 blockade (Hu5F9-G4) combined with rituximab has synergistic activity in an early phase clinical trial of DLBCL and follicular lymphoma (FL) [54].

MiRNAs are endogenous noncoding small RNAs participate in the occurrence and development of human malignancies. Recent studies have clarified that the specific miRNAs are involved in regulating the polarization direction and functional phenotype of TAMs. For example, miR-130, miR-33 and miR-155 can transform TAMs from the M2-like to M1-like phenotype [55, 56].

#### Targeting MDSCs

Considering the roles of MDSCs in hematological malignancies, it is reasonable to serve MDSCs as a promising target. Signal transducer and activator of transcription 3 (STAT3) and cyclooxygenase 2 (COX2)/PGE2 play a carcinogenic role in a variety of malignant tumors, which participate in the generation, maturation and accumulation of MDSCs [57]. The application of COX2 inhibitors could significantly reduce the abundance of MDSCs and block the function of MDSCs [58]. The results of a large population-based study demonstrated the survival advantages for newly diagnosed DLBCL patients who received COX2 inhibitor [59]. Emerging studies indicate that the phosphatidylinositol 3-kinase (PI3K)/AKT pathway participates in tumorigenesis by facilitating the immunosuppressive state of TME [60]. In HL, previous investigations have revealed that RP6530, a PI3Kδ/γ inhibitor, decreases the percentage of MDSCs, repolarizes TAMs to the M1-like phenotype and downregulates the expression of iNOS, thereby leading to tumor regression [61].

In addition, miRNAs could also affect the function of MDSCs. MiR-30a increases the immunosuppressive function of MDSCs by decreasing SOCS3 mRNA in B-cell lymphoma. Targeting miR-30a could reduce MDSC-mediated immunosuppressive and the number of MDSCs [62]. Li et al. reported that c-Rel, a novel immune checkpoint in MDSCs, participated in various processes, containing development, function and metabolism of MDSCs. Chen and colleagues developed R96A, a c-Rel inhibitor, which can significantly reduce the progression of lymphoma and synergistically enhance the response to anti-PD-1 antibodies [63].

#### Targeting TANs

Substantial studies have examined various compounds capable of modulating neutrophils [64]. Similar to the cases for TAMs, the combination of SIRPα and CD47 on TANs also mediates the immune escape. In Burkitt lymphoma (BL), KWAR23 (an anti-SIRPα antibody) was found to combine with SIRPα at high affinity and consequently increased the TANs-mediated phagocytosis of BL cells [65].

Inhibition of TANs during tumor progression serves as another effective strategy. In TME, it is implied that the CXCL12/CXCR4 axis plays a complex role in regulating the retention of TANs at inflammatory sites [66]. Emerging studies have revealed that AMD3100, an effective CXCR4 antagonist, could reverse migration and maintain the balance between bone marrow and peripheral blood, thereby inhibiting tumor growth and metastasis [67].

In addition, the CXCR2 axis is proved to be involved in the recruitment of N2 phenotype cells. DLBCL-derived IL-8 interacts with CXCR2 on TANs, forming neutrophil extracellular traps, and further activates the downstream pathway of Toll-like receptor 9 (TLR9) to boost the proliferation and migration of DLBCL cells. Nevertheless, preliminary evidence showed that deoxyribonuclease I, neutrophil elastase inhibitor and blocking CXCR2 or TLR9 could restrain the progression of DLBCL [68]. Noteworthy, potential cautions and risks are still existing, such as intolerable adverse effects and infections.

#### NK cell-based immunotherapy

Recently, NK cell-based therapy has evolved to become the major area of immunotherapy, and it may complement the limitations of T cell-based therapy [35]. The function of NK cells could be restored by blockade of checkpoint inhibitors. Natural killer group 2A (NKG2A), a type II membrane receptor, inhibits NK cells by binding to HLA-E in CLL [69]. Therefore, blocking NKG2A can effectively restore the function of NK cells. In addition, killer cell immunoglobulin-like receptors (KIRs) inhibit the activation of NK cells by binding to HLA-C. Lirilumab, an anti-KIR monoclonal antibody (mAb), could enhance the effect of rituximab and the spontaneous cytotoxicity of NK cells by restraining the binding of KIRs to MHC-I antigen in B-cell lymphoma [70]. Furthermore, blockade of other inhibitory receptors, such as T cell immunoreceptor with immunoglobulin and ITIM domain, T cell immunoglobulin mucin receptor 3 and PD-1, has shown promising potential for NK cell-based immunotherapy [71].

Direct enhancement of NK cell activity is considered another type of NK cell-based therapy. Cytokines, such as IL-2 or IL-15, could stimulate the activation and proliferation of NK cells. Therefore, it is necessary to provide NK cells with survival factors to increase their persistence in vivo [72]. NK cell-based therapy shows advantages over targeting T cells in some respects, providing an alternative immunotherapeutic method for T cell-based therapy.

#### Targeting CAFs

Given the tumor-promoting effect of CAFs, targeting CAFs alone or in combination with other immunotherapies has emerged as a promising therapeutic approach [73]. Strategies targeting CAFs are currently being explored, including (1) locating and depleting CAFs via cell surface markers, (2) targeting related signaling pathways downstream effectors, (3) restoring activated CAFs and (4) targeting CAF-derived ECM proteins and associated signaling [74]. Although many strategies have been explored, clinical success is still lacking in B-cell lymphoma, which may be related to the heterogeneity and lack specific biomarkers of CAFs [75].

#### Targeting the ECM

MMPs, enzymes targeting the ECM that cause collagen degradation, could delay the process of tissue regeneration and influence the survival, expansion and progression of tumors [76]. MMP-9, one type of MMPs, has been proved to be involved in the angiogenesis of NHL [77]. In DLBCL, the M2 TAMs could promote tumor progression by inducing cleavage of ECM via legumain [78]. As the ECM can act as a barrier to prevent effective drug delivery, targeting the ECM is expected to overcome therapy resistance by improving effective drug delivery [79]. In addition, the ECM is also considered to play a critical role in the survival and maintenance of cancer stem cells.

#### Targeting other TME cells

Furthermore, targeting other TME cells also provides novel insights for clinical treatment. Personalized DC vaccines display great potential in facilitating efficient and targeted tumor immunotherapy by activating T cell immunity and suppressing Tregs [80]. In terms of eosinophils, ample evidence indicates that they playing significant role in the TME and influence the response to therapy in malignancies. Eosinophils can release chemokines, such as IFN-γ, which are essential for antagonization of CTLA-4-induced vessel normalization, thereby promoting angiogenesis [81]. However, as increasing number of patients are treated with these new regimens, longer follow-up is needed to determine the clinical value of targeting eosinophils for cancer treatment. Targeting monocytes may also prolong survival and provide higher rates of response for certain patients because monocyte-derived TAMs are enriched in genes correlated with immunosuppression, indicating their contribution to tumor progression [82].

### Targeting hypoxia, a hallmark of the TME

**Fig. 3:**
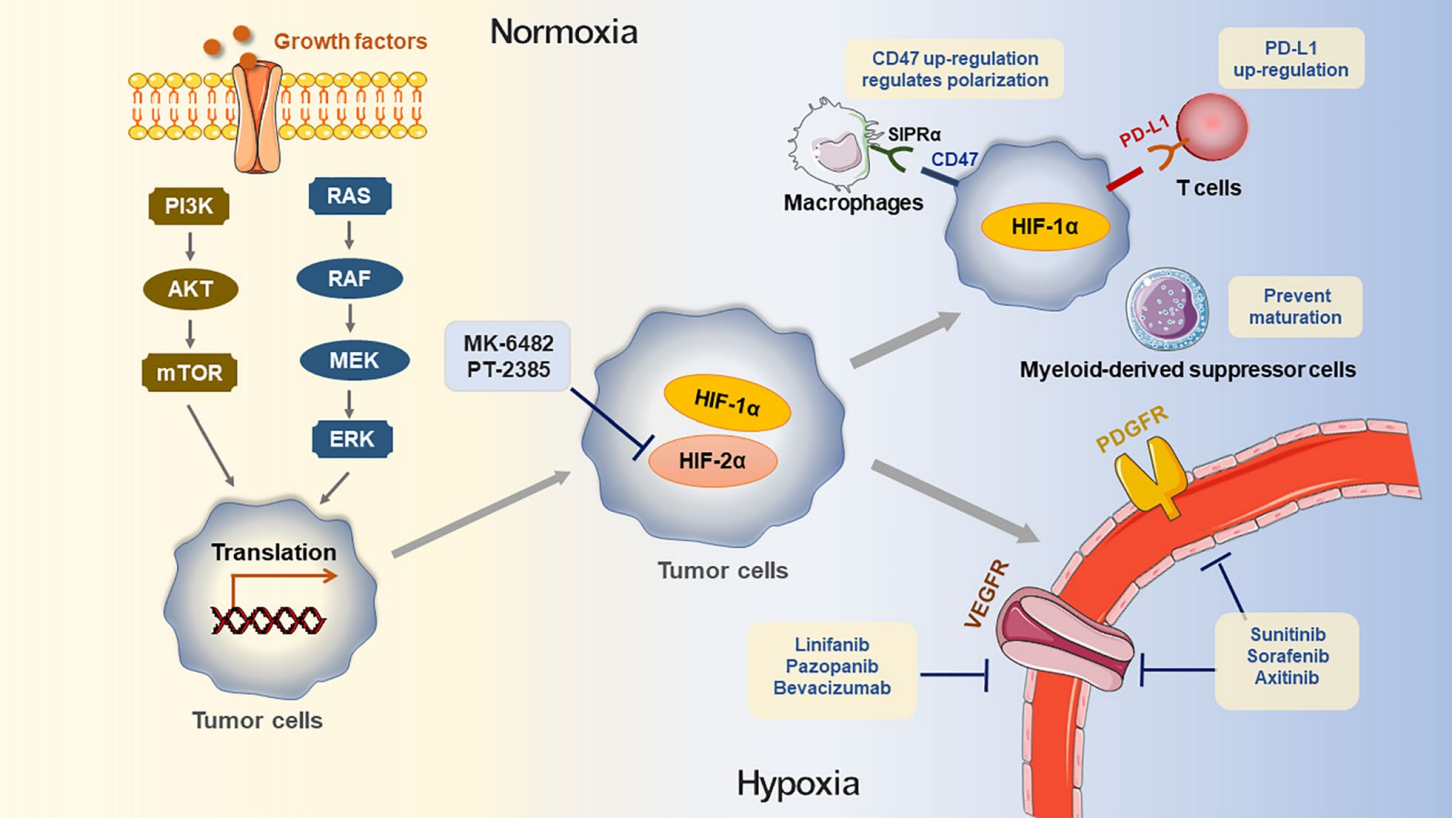
Impact of hypoxic TME and targeted therapy. Growth factors regulate HIF-1α through MAPK/ERK and PI3K/AKT/mTOR pathway, consequently induce the simulation of HIF-1α mRNA transactivation. Under the hypoxic microenvironment, HIF-1α in cells up-regulates the expression of PD-L1 in hypoxic tumor cells, prevents MDSC from maturation at the tumor site and involves the up-regulation of CD47 on the surface of tumor cells. After CD47 combines with SIRPα on the surface of macrophages, tumor cells provide a robust “don't phagocytize me” signal, thus preventing the phagocytosis of macrophages

Due to the rapid growth of tumors, most of the TME is characterized and affected by hypoxia. Hypoxia could induce TAM polarization [83], promote the accumulation of MDSCs and Tregs [84] and downregulate the activity of NK cells (Fig. 3) [85]. Hypoxia inducible factor-1 (HIF-1) is a transcription factor composed of HIF-1α and HIF-1β. HIF-1α synthesis is regulated by PI3K or mitogen-activated protein kinase (MAPK) signal pathways [86]. High expression of HIF-1α induces tumor cells to adapt to hypoxia and grow rapidly [87]. In MCL, HIF-1α participates in the downregulation of BACH2, which not only accelerates tumor formation but also promotes tumor spread to the spleen and bone marrow [88]. It has been reported that HIF-1α induces the expression of hexokinase II to promote the development of B-cell lymphoma, which provides an innovative perspective for targeted therapy [89]. Through the vascular endothelial growth factor A (VEGFA)/vascular endothelial growth factor receptor 1 (VEGFR1) axis, HIF-1α contributes to angiogenesis and promotes lymphoma cells to resist apoptosis [90].

On the other hand, HIF-2α has been indicated to promote the survival of hypoxic cells [91]. MK-6482 and PT2385, inhibitors of HIF-2α, are currently applied in renal cell carcinoma [92, 93] and capable of achieving desirable therapeutic effects. Further investigations are needed to explore the efficacy of targeting hypoxia for the treatment of B-cell lymphoma.

### Immunotherapies for reversion of T cell exhaustion

#### PD-1/programmed cell death ligand 1 (PD-L1)

PD-1/PD-L1 is a negative modulatory signaling pathway that leads to the exhaustion of T cell immunity. Under normal circumstances, the combination of PD-1 and PD-L1 on cytotoxic T lymphocytes (CTLs) controls excessive immunity. However, many tumor cells also express PD-L1 to protect themselves [94]. The PD-1/PD-L1 blockade restores T cells to an activated state and elicits more pronounced antitumor effects by rescuing exhausted T cells through JAK/STAT and PI3K/AKT pathways [95, 96]. In the management of B-cell lymphoma, the PD-1/PD-L1 blockade therapy has shown potential effects (Table 3). Recent studies have illuminated that a novel antibody (YM101) blocking TGF-β and PD-1/PD-L1 pathways and promoting the formation of "hot" immune-inflamed TME, shows better antitumor effects than single anti-PD-L1 antibody treatment [97]. Moreover, combination with antibody–drug conjugates could be a feasible option for relapsed/refractory (R/R) B-NHL, which exhibits advantages over the use of PD-1 inhibitor alone [98].

**Table 3:** Results from clinical trials of PD-1/PD-L1 blockade in B-cell lymphoma

Identifier	B-cell lymphoma	Antibody, dose	Clinical significance	References
*Anti-PD-1 monoclonal antibody*				
NCT02332668	R/R HL (n = 15)	Pembrolizumab 2 mg/kg q3w	ORR 60%, CR 13%, PR 47%	[99]
NCT03155425	R/R cHL (n = 75)	Camrelizumab 200 mg q2w	ORR 76%, CR 28%, PR 48%	[100]
NCT02961101 NCT03250962	R/R cHL (n = 86)	Camrelizumab 200 mg q3w or Decitabine 10 mg/d, d1-5 + Camrelizumab 200 mg, d8 q3w	Camrelizumab (n = 19): CR 32% Decitabine + Camrelizumab (n = 42): CR 71%	[101]
NCT03114683	R/R cHL (n = 96)	Sintilimab 200 mg q3w	ORR 80.4%, CR 34%, PR 47%	[102]
NCT03209973	R/R cHL (n = 70)	Tislelizumab 200 mg q3w	ORR 87.1%, CR 62.9% 9 months: PFS 74.5%	[103]
NCT01953692	R/R PMBCL (n = 21)	Pembrolizumab 10 mg/kg q2w (n = 10) 200 mg q3w (n = 11)	ORR 48%, CR 33%, PR 14%	[104]
NCT02332980	R/R CLL (n = 16) RT DLBCL (n = 9)	Pembrolizumab 200 mg q3w	ORR 16% (CLL:0%, DLBCL:44%) CR (DLBCL) 11%, PR (DLBCL) 22%	[105]
*Anti-PD-L1 monoclonal antibody*				
NCT02401048	R/R FL (n = 27) R/R DLBCL (n = 34)	Ibrutinib 560 mg qd + Durvalumab 10 mg/kg q2w	ORR (all patients) 25% ORR (FL) 26% ORR (GCB DLBCL) 13% ORR (non‐GCB DLBCL) 38% CR (FL) 4% CR (GCB DLBCL) 6% CR (non-GCB DLBCL) 31% median PFS 4.6 months median OS 18.1 months	[106]
NCT02541604	HL (n = 9) NHL (n = 3)	Atezolizumab 15 mg/kg (≤ 18 years) Atezolizumab 1200 mg (18-29 years)	PR (HL) 22.2% PR (NHL) 33.3%	[107]

However, the clinical applications of PD-1/PD-L1 blockade may be hindered by the decreased proportion of responding patients and acquired resistance during treatment. Recent studies have demonstrated that PD-1 blockade combined with anti-CD20 mAbs could deplete lymphoma cells, reshape the TME and achieve long-term antitumor responses by inducing immune cell infiltration [108]. Interestingly, the heterogeneity of the TME makes the efficacy of PD-1/PD-L1 blockade different. “Hot” TME is associated with high infiltration of tumor infiltration lymphocytes (TILs) and accompanied by higher levels of IFN, which may indicate the potential of therapy benefits. In contrast, noninflammatory tumors with a “cold” TME may respond poorly to PD-1/PD-L1 blockade [109]. Therefore, the abundance of TILs also acts as a potential predictor to forecast the efficacy of PD-1/PD-L1 inhibitors [110].

#### CTLA-4

CTLA-4 is a negative immunomodulator and constitutively expressed on Tregs [111]. CTLA-4 provides inhibitory signals to T cells and shares B7 ligands, such as B7-1 (CD80) and B7-2 (CD86), with CD28. Compared with CD28, CTLA-4 presents a higher affinity [112]. Different from PD-1 blockade, which relieves the immunosuppression of T cells, anti-CTLA-4 therapies mainly enhance T cell activation and proliferation by downregulating the immune suppression mediated by Tregs [113]. They selectively remove Tregs from tumors through antibody-dependent cell-mediated cytotoxicity and deplete Tregs via an Fc-dependent mechanism [114]. Ingram et al. found that administration of H11 without Fc fragment-mediated CTLA-4 blockade significantly attenuated the antitumor effect [115]. Collectively, anti-CTLA-4 agents need to modify the Fc domain of mAbs to deplete Tregs for therapeutic effects.

Results from recent clinical trials have shown that CTLA-4 alone or in combination with other agents exert a positive effect in the treatment of melanoma [116], but further investigations of the application in B-cell lymphoma are warranted [117]. Patel et al. observed that CTLA-4 was the main checkpoint protein within the classical HL (cHL) TME, and Hodgkin Reed–Sternberg cells exploited the interaction between CTLA-4 and CD86 as an escape pathway [118]. Emerging evidence suggests that anti-CTLA-4 agents synergizes with anti-CD20 mAbs in treating R/R B-cell lymphoma [119].

#### Chimeric antigen receptor (CAR)-T cells

T cells can be engineered to express a CAR to target certain cancer cells. Generally, the CAR consists of three parts: (1) a single-chain antibody fragment (sc Fγ) that is responsible for recognizing the antigen; (2) a transmembrane region that connects the transition region inside and outside of the cell; and (3) an intracellular signal domain that transmits TCR-like signaling to cell when combining with antigen outside of the cell [120]. CARs could locate and recognize tumor cells and release various effectors, effectively killing tumor cells and consequently achieving the purpose of treating malignant tumors [121]. Four CAR-T cell products currently approved by FDA are summarized in Table 4.

**Table 4:** Results of currently approved CAR-T cell products for B-cell lymphoma

Cell product	Target	Cell dose	Indication	Clinical significance	Complications	References
Tisagenlecleucel (Kymriah)	CD19	0.1–6 × 10^8^	R/R DLBCL	ORR 52%, CR 40%, PR 12%	Cytopenias:32% Infections:20% NEs (grade 3 or worse):12%, CRS (grade 3 or worse):22%	[122]
Axicabtagene ciloleucel (Yescarta)	CD19	2 × 10^6^/kg	R/R large B-cell lymphoma	ORR 83%, CR 58%	Pyrexia:87% Anaemia:68% NEs (grade 3 or worse):32% CRS (grade 3 or worse):11%	[123]
		2 × 10^6^/kg	R/R FL	ORR 95%, CR 80%	NEs (grade 3 or worse):19% CRS (grade 3 or worse):11%	[124]
Brexucabtagene Autoleucel (Tecartus)	CD19	2 × 10^6^/kg	R/R MCL	ORR 85%, CR 58%	Cytopenias:94% Infections:32% NEs (grade 3 or worse):31%, CRS (grade 3 or worse):15%	[125]
Lisocabtagene maraleucel (Breyanzi)	CD19	50/100/150 × 10^6^	R/R large B-cell lymphoma	ORR 73%, CR 53%	Neutropenia:60% Anaemia:37% Thrombocytopenia:27% NEs (grade 3 or worse):10%, CRS (grade 3 or worse):2%	[126]

Although CAR-T cell therapies targeting CD19, CD22, CD30 and so on have revealed significant clinical effects in hematological malignancies [127–129], the senescence and exhaustion of T cells negatively influence the effect. It was confirmed that engineered CAR-T cells to overexpress c-Jun could decrease or displace AP-1i from chromatin, thereby preventing T cell exhaustion [130]. Agonists of T cell receptors, 4-1BB and OX40, could provide another stimulatory signal for CAR-T cells, as they increase the activity of T cells and make CAR-T cells resistant to the suppressive effects of TME [131]. Moreover, the urokinase-type plasminogen activator receptor-specific CAR-T cells have emerged as a novel insights due to it is capable of ablating senescent cells [132].

With further investigations on CAR-T cell therapies, strategies targeting nontumor components of TME have also been proposed. Heparan sulfate proteoglycans (HSPG) is an integral component of the ECM, and heparanase (HPSE) degrades HSPGs. Therefore, to expedite CAR-T cell penetration into the tumor stroma, the researchers have designed CAR-T cells overexpressing HPSE to degrade HSPG [133]. Fibroblast activation protein (FAP) could modify the ECM by changing fibronectin orientation, and the FAP-redirected CAR-T cells have been established to deplete CAFs expressing FAP. These armored CAR-T cells decrease tumor vascular density, disrupt the spatial orientation of tumor cells and inhibit the genesis of tumor stroma, which may exert profound effects on tumor growth [134]. TAMs expressing folate receptor β (FRβ) are similar to M2 macrophages. FRβ-specific CAR-T cells reprogram the TME and significantly delay tumor progression by depleting immunosuppressive TAMs [135]. CAR-T cells that target tumor vasculature, metabolism, hypoxia, and other immunosuppressive cells and cytokines also show great clinical potential [136].

The antitumor efficacy of other immune cells, such as macrophages, NK cells and Tregs, are explored as well [137]. It has been reported that genetically engineered macrophages with CAR could enhance phagocytic ability, induce M2 cell transform into the M1 phenotype, resist the effects of immunosuppressive cytokines and strengthen the antitumor activity of T cells [138]. Liu et al. found that CAR-NK cells could partly overcome the toxic effects of CAR-T cells and reduce the incidence of severe cytokine release syndrome and related neurologic toxic effects [139]. Zhang et al. reported that the induced pluripotent stem cells could supply an infinite source for CAR-expressing macrophages, which have enhanced antitumor effects both in vitro and in vivo [140].

### TME-mediated drug resistance

Although the mechanisms of drug resistance induced by heterogeneity within the TME is distinct, some studies suggest that the TME is crucial in drug resistance, resulting in limited clinical benefit for patients with cancer [141]. A variety of factors contribute to determining the clinical responses, and distinguishing the three basic immune profiles is essential to enhance the patients' responses to immunotherapy. (1) Inflamed TME: There are enormous numbers of infiltrating immune cells (especially CD8^+^ T cells), high expression of PD-L1 and high level of inflammatory cytokines. The reason for drug resistance may be that T cells express other inhibitory immune checkpoints [142, 143]. In this regard, a combination of immune checkpoint inhibitors could be selected. Emerging discoveries indicated that the combination of CTLA-4 and PD-1 blockers increased response rates and efficacy [144]. (2) Immune-excluded TME: The process of effector T cells infiltration is blocked by tumor blood vessels, stroma and other physical barriers, eventually leading to drug resistance [142]. As a key barriers of immune infiltration into the TME, Tregs exclude pro-inflammatory cells from the TME and limit the activity of effector cells [145]. (3) Noninflamed TME (immune-desert TME): Such a TME is characterized by the presence of immunosuppressive cells and the absence of immune cells in the tumor tissue [142]. Hence, transforming a “cold” (noninflamed) into a “hot” (inflamed) TME could be an ideal way to facilitate the infiltration, activation and proliferation of effector T cells [146].

### TME characteristics with prognostic utility

Several studies have discovered that the composition of TME cells is highly relevant to the prognosis of B-cell lymphomas, such as cHL, DLBCL and FL [147]. Generally, CD163 is a biomarker of TAMs (M2 phenotype) in malignant tumors. It was reported that a higher level of CD163^+^ and a higher CD163^+^/CD68^+^ cell ratio were linked to poorer progression-free survival (PFS) and overall survival (OS) in DLBCL [148]. High expression levels of LAG3 in TAM-rich regions are associated with poorer OS [149]. It is reported that in high-grade lymphomas, like DLBCL and BL, TANs produce a proliferation-inducing ligand, the high expression of which is closely related to inferior OS [150]. In FL and DLBCL, increased NK cell infiltration is always associated with a favorable prognosis [18]. A recent study proposed a novel prognostic risk model based on eosinophil counts. The results suggest that eosinophil counts facilitate anti-CD19 CAR-T cell therapy and positively correlate with clinical outcomes in B-NHL [151].

A series of studies have proved that the prognostic roles of the expression of PD-1 and PD-L1 remain unclear. Many investigators have found that PD-L1 expression is related to an inferior prognosis. Kiyasu et al. suggested that PD-L1^+^ tumor cells had shortened survival in patient with DLBCL, compared to those with PD-L1^−^ tumor cells [152]. A meta-analysis supported that PD-L1 expression in tumor cells of DLBCL patients was significantly correlated with poor prognosis [153]. However, Kwon et al. reported no prognostic significance of PD-L1 expression in DLBCL patients treated with R-CHOP [154]. Additionally, Ishikawa et al. demonstrated that PD-L1 expression on microenvironment immune cells was strongly associated with better OS in patients with DLBCL [155]. Likewise, Pollari et al. reported that high expressions levels of PD-L1 on TAMs and TILs were related to prolonged survival in primary testicular lymphoma [156].

### TME of B-cell lymphoma in the era of novel technology

#### Single-cell RNA sequencing (scRNA-seq)

ScRNA-seq, a novel technology, could be used to comprehensively analyze cell type-specific transcriptomic changes, thereby deepening the understanding of heterogeneity in the TME [157]. The development of scRNA-seq has significantly improved the ability to overview specific genes, but it cannot detect spatial information. The recently developed spatial transcriptome method can compensate for this shortcoming, which will facilitate the understanding of tumorigenesis and progression [158]. The databases with cancer scRNA-seq datasets for decoding and visualizing the functional status of cancer cells have been established, such as SC2disease [159], CancerSEA [160], Vascular Single Cells [161], PanglaoDB [162], Cell Marker [163], Animal Cell Atlas [164] and Alona [165].

#### Nanoparticle (NP)-based immunotherapies

Due to the biological characteristics of NPs, they can be used to accurately deliver drugs [166]. The regulatory effects of NPs on the TME are reflected in the following aspects: (1) relieving the immunosuppressive TME with NPs modified by various ligands, (2) delivering tumor-associated antigens and adjuvants to stimulate antigen-presenting cells through NPs, thus enhancing the antitumor immune response, and (3) affecting the abnormal structure of the TME and reshaping the tumor immune microenvironment with NPs [167]. Notably, before nano-immunotherapy becomes a large-scale clinical strategy, researchers need to be cautious about keeping the balance between therapeutic benefit and toxicity risks. Owing to their superparamagnetic properties and high surface-to-volume ratios, the novel nanomaterials are engineered in clinical applications by enhancing the specificity of chemotherapy and controlling the release speed of drugs [168]. The NPs are considered to be an emerging dimension of immunotherapy research.

#### Other novel technologies

Given that there are numerous tumor-associated mutations and phenotypic variations in tumors, explorations of targeting the TME with genetic therapies and armed oncolytic viruses could promote the tumor response or restrain tumor tolerance [169]. For instance, the hyaluronidase-armed oncolytic virus could degrade the hyaluronan-rich matrix in an attempt to improve virus penetration and inhibit tumor growth in xenograft models. A phase I clinical study also supports that hyaluronidase-armed oncolytic viruses could modulate the TME more pro-inflammatory and alleviate potential toxicity and unwanted cytokine release [170]. TME–targeted therapies in combination with immunotherapies have emerged as a promising approach for cancer treatment. Modified second-generation CAR-T cells could remodel the immunosuppressive TME and revive exhausted T cells, which may further improve clinical efficacy [7].

## Conclusions

In this review, we systematically summarize that the composition of the TME plays a vital role in various processes, including the progression, treatment, drug resistance and prognosis of B-cell lymphoma. Targeting TME components is expected to provide novel insights for the precise treatment of B-cell lymphoma. Nevertheless, there are still many unresolved issues, such as drug resistance and the feasibility of drug combination. Further studies are warranted to verify and promote the clinical applications of TME-based targeted therapy. A deep understanding of the contribution of the TME to B-cell lymphomas will help us provide patients with more feasible and effective treatment strategies.

## Data Availability

Not applicable.

## Abbreviations

Arg1: Arginase1
BL: Burkitt lymphoma
CAFs: Cancer-associated fibroblasts
CAR: Chimeric antigen receptor
cHL: Classical Hodgkin lymphoma
CLL: Chronic lymphocytic leukemia
COX2: Cyclooxygenase 2
CSF-1: Colony-stimulating factor-1
CSF-1R: CSF-1 receptor
CTLs: Cytotoxic T lymphocytes
CTLA-4: Cytotoxic T-lymphocyte-associated antigen 4
DCs: Dendritic cells
DLBCL: Diffuse large B-cell lymphoma
ECM: Extracellular matrix
FAP: Fibroblast activation protein
FL: Follicular lymphoma
FRβ: Folate receptor β
HIF: Hypoxia inducible factor
HL: Hodgkin lymphoma
HPSE: Heparanase
HSPG: Heparan sulfate proteoglycan
IFN: Interferon
IL: Interleukin
iNOS: Inducible nitric oxide synthase
KIRs: Killer cell immunoglobulin-like receptors
M: Monocytic
mAbs: Monoclonal antibodies
MAPK: Mitogen-activated protein kinase
MCL: Mantle cell lymphoma
MDSCs: Myeloid-derived suppressor cells
MHC-I: Major histocompatibility complex class I
MiRNAs: MicroRNAs
MMPs: Matrix metalloproteinases
NHL: Non-Hodgkin lymphoma
NK: Natural killer
NKG2A: Natural killer cell group 2A
NKG2D: Natural killer cell group 2D
NLCs: Nurse-like cells
NP: Nanoparticle
OS: Overall survival
PD-1: Programmed cell death 1
PD-L1: Programmed cell death ligand 1
PFS: Progression-free survival
PGE2: Prostaglandin E2
PI3K: Phosphatidylinositol 3-kinase
PMN: Polymorphonuclear
ROS: Reactive oxygen species
R/R: Relapsed/refractory
scRNA-seq: Single-cell RNA sequencing
STAT3: Signal transducer and activator of transcription 3
TAMs: Tumor-associated macrophages
TANs: Tumor-associated neutrophils
TILs: Tumor infiltration lymphocytes
TLR9: Toll-like receptor 9
TME: Tumor microenvironment
TGF-β: Transforming growth factor-β
Tregs: Regulatory T cells
VEGFA: Vascular endothelial growth factor A
VEGFR1: Vascular endothelial growth factor receptor 1

## References

[1] Ennishi D, Hsi ED, Steidl C, Scott DW (2020). Toward a new molecular taxonomy of diffuse large B-cell lymphoma. Cancer Discov.

[2] Sehn LH, Salles G (2021). Diffuse large B-cell lymphoma. N Engl J Med.

[3] Casey S, Amedei A, Aquilano K, Azmi A, Benencia F, Bhakta D, Bilsland A, Boosani C, Chen S, Ciriolo M, Crawford S, Fujii H, Georgakilas A, Guha G, Halicka D, Helferich W, Heneberg P, Honoki K, Keith W, Kerkar S, Mohammed S, Niccolai E, Nowsheen S, Vasantha Rupasinghe H, Samadi A, Singh N, Talib W, Venkateswaran V, Whelan R, Yang X (2015). Cancer prevention and therapy through the modulation of the tumor microenvironment. Semin Cancer Biol.

[4] Wang L, Ding K, Zheng C, Xiao H, Liu X, Sun L, Omer R, Feng Q, Zhang Z (2020). Detachable nanoparticle-enhanced chemoimmunotherapy based on precise killing of tumor seeds and normalizing the growing soil strategy. Nano Lett.

[5] Hui L, Chen Y (2015). Tumor microenvironment: sanctuary of the devil. Cancer Lett.

[6] Junttila M, de Sauvage F (2013). Influence of tumour micro-environment heterogeneity on therapeutic response. Nature.

[7] Bejarano L, Jordāo M, Joyce J (2021). Therapeutic targeting of the tumor microenvironment. Cancer Discov.

[8] Steidl C, Lee T, Shah S, Farinha P, Han G, Nayar T, Delaney A, Jones S, Iqbal J, Weisenburger D, Bast M, Rosenwald A, Muller-Hermelink H, Rimsza L, Campo E, Delabie J, Braziel R, Cook J, Tubbs R, Jaffe E, Lenz G, Connors J, Staudt L, Chan W, Gascoyne R (2010). Tumor-associated macrophages and survival in classic Hodgkin's lymphoma. N Engl J Med.

[9] Taskinen M, Karjalainen-Lindsberg M, Nyman H, Eerola L, Leppä S (2007). A high tumor-associated macrophage content predicts favorable outcome in follicular lymphoma patients treated with rituximab and cyclophosphamide-doxorubicin-vincristine-prednisone. Clin Cancer Res.

[10] Wu K, Lin K, Li X, Yuan X, Xu P, Ni P, Xu D (2020). Redefining tumor-associated macrophage subpopulations and functions in the tumor microenvironment. Front Immunol.

[11] Marini O, Spina C, Mimiola E, Cassaro A, Malerba G, Todeschini G, Perbellini O, Scupoli M, Carli G, Facchinelli D, Cassatella M, Scapini P, Tecchio C (2016). Identification of granulocytic myeloid-derived suppressor cells (G-MDSCs) in the peripheral blood of Hodgkin and non-Hodgkin lymphoma patients. Oncotarget.

[12] Azzaoui I, Uhel F, Rossille D, Pangault C, Dulong J, Le Priol J, Lamy T, Houot R, Le Gouill S, Cartron G, Godmer P, Bouabdallah K, Milpied N, Damaj G, Tarte K, Fest T, Roussel M (2016). T-cell defect in diffuse large B-cell lymphomas involves expansion of myeloid-derived suppressor cells. Blood.

[13] Gabrilovich D (2017). Myeloid-derived suppressor cells. Cancer Immunol Res.

[14] Jaillon S, Ponzetta A, Di Mitri D, Santoni A, Bonecchi R, Mantovani A (2020). Neutrophil diversity and plasticity in tumour progression and therapy. Nat Rev Cancer.

[15] Manfroi B, Moreaux J, Righini C, Ghiringhelli F, Sturm N, Huard B (2018). Tumor-associated neutrophils correlate with poor prognosis in diffuse large B-cell lymphoma patients. Blood Cancer J.

[16] Marcheselli R, Bari A, Tadmor T, Marcheselli L, Cox M, Pozzi S, Ferrari A, Baldini L, Gobbi P, Aviv A, Pugliese G, Federico M, Polliack A, Sacchi S (2017). Neutrophil-lymphocyte ratio at diagnosis is an independent prognostic factor in patients with nodular sclerosis Hodgkin lymphoma: results of a large multicenter study involving 990 patients. Hematol Oncol.

[17] Cozar B, Greppi M, Carpentier S, Narni-Mancinelli E, Chiossone L, Vivier E (2020). Tumor-infiltrating natural killer cells. Cancer Discov.

[18] Klanova M, Oestergaard M, Trněný M, Hiddemann W, Marcus R, Sehn L, Vitolo U, Bazeos A, Goede V, Zeuner H, Knapp A, Sahin D, Spielewoy N, Bolen C, Cardona A, Klein C, Venstrom J, Nielsen T, Fingerle-Rowson G (2019). Prognostic impact of natural killer cell count in follicular lymphoma and diffuse large B-cell lymphoma patients treated with immunochemotherapy. Clin Cancer Res.

[19] Han C, Liu T, Yin R (2020). Biomarkers for cancer-associated fibroblasts. Biomark Res.

[20] Bankov K, Doring C, Ustaszewski A, Giefing M, Herling M, Cencioni C, Spallotta F, Gaetano C, Kuppers R, Hansmann ML, Hartmann S (2019). Fibroblasts in nodular sclerosing classical hodgkin lymphoma are defined by a specific phenotype and protect tumor cells from brentuximab-vedotin induced injury. Cancers (Basel).

[21] Haro M, Orsulic S (2018). A paradoxical correlation of cancer-associated fibroblasts with survival outcomes in B-cell lymphomas and carcinomas. Front Cell Dev Biol.

[22] Staiger A, Duppel J, Dengler M, van der Kuip H, Vöhringer M, Aulitzky W, Rosenwald A, Ott G, Horn H (2017). An analysis of the role of follicular lymphoma-associated fibroblasts to promote tumor cell viability following drug-induced apoptosis. Leuk Lymphoma.

[23] Ciavarella S, Vegliante M, Fabbri M, De Summa S, Melle F, Motta G, De Iuliis V, Opinto G, Enjuanes A, Rega S, Gulino A, Agostinelli C, Scattone A, Tommasi S, Mangia A, Mele F, Simone G, Zito A, Ingravallo G, Vitolo U, Chiappella A, Tarella C, Gianni A, Rambaldi A, Zinzani P, Casadei B, Derenzini E, Loseto G, Pileri A, Tabanelli V (2019). Dissection of DLBCL microenvironment provides a gene expression-based predictor of survival applicable to formalin-fixed paraffin-embedded tissue. Ann Oncol.

[24] Sugimoto T, Watanabe T (2016). Follicular lymphoma: the role of the tumor microenvironment in prognosis. J Clin Exp Hematopathol.

[25] Janco JMT, Lamichhane P, Karyampudi L, Knutson KL (2015). Tumor-infiltrating dendritic cells in cancer pathogenesis. J Immunol.

[26] Pathria P, Louis TL, Varner JA (2019). Targeting tumor-associated macrophages in cancer. Trends Immunol.

[27] Lin Y, Xu J, Lan H (2019). Tumor-associated macrophages in tumor metastasis: biological roles and clinical therapeutic applications. J Hematol Oncol.

[28] Mantovani A, Marchesi F, Malesci A, Laghi L, Allavena P (2017). Tumour-associated macrophages as treatment targets in oncology. Nat Rev Clin Oncol.

[29] Wang J, Li D, Cang H, Guo B (2019). Crosstalk between cancer and immune cells: role of tumor-associated macrophages in the tumor microenvironment. Cancer Med.

[30] Zhou J, Nefedova Y, Lei A, Gabrilovich D (2018). Neutrophils and PMN-MDSC: their biological role and interaction with stromal cells. Semin Immunol.

[31] Tian X, Shen H, Li Z, Wang T, Wang S (2019). Tumor-derived exosomes, myeloid-derived suppressor cells, and tumor microenvironment. J Hematol Oncol.

[32] Ren W, Zhang X, Li W, Feng Q, Feng H, Tong Y, Rong H, Wang W, Zhang D, Zhang Z, Tu S, Zhu X, Zhang Q (2019). Exosomal miRNA-107 induces myeloid-derived suppressor cell expansion in gastric cancer. Cancer Manag Res.

[33] Masucci M, Minopoli M, Carriero M (2019). Tumor associated neutrophils. Their role in tumorigenesis, metastasis, prognosis and therapy. Front Oncol.

[34] Giese M, Hind L, Huttenlocher A (2019). Neutrophil plasticity in the tumor microenvironment. Blood.

[35] Myers JA, Miller JS (2021). Exploring the NK cell platform for cancer immunotherapy. Nat Rev Clin Oncol.

[36] Crinier A, Narni-Mancinelli E, Ugolini S, Vivier E (2020). SnapShot: natural killer cells. Cell.

[37] Ping Q, Yan R, Cheng X, Wang W, Zhong Y, Hou Z, Shi Y, Wang C, Li R (2021). Cancer-associated fibroblasts: overview, progress, challenges, and directions. Cancer Gene Ther.

[38] Sahai E, Astsaturov I, Cukierman E, DeNardo D, Egeblad M, Evans R, Fearon D, Greten F, Hingorani S, Hunter T, Hynes R, Jain R, Janowitz T, Jorgensen C, Kimmelman A, Kolonin M, Maki R, Powers R, Puré E, Ramirez D, Scherz-Shouval R, Sherman M, Stewart S, Tlsty T, Tuveson D, Watt F, Weaver V, Weeraratna A, Werb Z (2020). A framework for advancing our understanding of cancer-associated fibroblasts. Nat Rev Cancer.

[39] De Jaeghere EA, Denys HG, De Wever O (2019). Fibroblasts fuel immune escape in the tumor microenvironment. Trends Cancer.

[40] Mhaidly R, Mechta-Grigoriou F (2021). Role of cancer-associated fibroblast subpopulations in immune infiltration, as a new means of treatment in cancer. Immunol Rev.

[41] Walker C, Mojares E, Del Río HA (2018). Role of extracellular matrix in development and cancer progression. Int J Mol Sci.

[42] Baghban R, Roshangar L, Jahanban-Esfahlan R, Seidi K, Ebrahimi-Kalan A, Jaymand M, Kolahian S, Javaheri T, Zare P (2020). Tumor microenvironment complexity and therapeutic implications at a glance. Cell Commun Signal.

[43] Bonnans C, Chou J, Werb Z (2014). Remodelling the extracellular matrix in development and disease. Nat Rev Mol Cell Biol.

[44] Pickup M, Mouw J, Weaver V (2014). The extracellular matrix modulates the hallmarks of cancer. EMBO Rep.

[45] Olingy C, Dinh H, Hedrick C (2019). Monocyte heterogeneity and functions in cancer. J Leukoc Biol.

[46] Tran Janco J, Lamichhane P, Karyampudi L, Knutson K (2015). Tumor-infiltrating dendritic cells in cancer pathogenesis. J Immunol (Baltimore, Md: 1950).

[47] Wu T, Dai Y (2017). Tumor microenvironment and therapeutic response. Cancer Lett.

[48] Petty AJ, Yang Y (2019). Tumor-associated macrophages in hematologic malignancies: new insights and targeted therapies. Cells.

[49] Papin A, Tessoulin B, Bellanger C, Moreau A, Le Bris Y, Maisonneuve H, Moreau P, Touzeau C, Amiot M, Pellat-Deceunynck C, Le Gouill S, Chiron D (2019). CSF1R and BTK inhibitions as novel strategies to disrupt the dialog between mantle cell lymphoma and macrophages. Leukemia.

[50] Polk A, Lu Y, Wang T, Seymour E, Bailey N, Singer J, Boonstra P, Lim M, Malek S, Wilcox R (2016). Colony-stimulating factor-1 receptor is required for nurse-like cell survival in chronic lymphocytic leukemia. Clin Cancer Res.

[51] DeNardo D, Ruffell B (2019). Macrophages as regulators of tumour immunity and immunotherapy. Nat Rev Immunol.

[52] Huang Y, Cai K, Xu P, Wang L, Huang C, Fang Y, Cheng S, Sun X, Liu F, Huang J, Ji M, Zhao W (2021). CREBBP/EP300 mutations promoted tumor progression in diffuse large B-cell lymphoma through altering tumor-associated macrophage polarization via FBXW7-NOTCH-CCL2/CSF1 axis. Signal Transduct Target Ther.

[53] Eladl E, Tremblay-LeMay R, Rastgoo N, Musani R, Chen W, Liu A, Chang H (2020). Role of CD47 in hematological malignancies. J Hematol Oncol.

[54] Advani R, Flinn I, Popplewell L, Forero A, Bartlett N, Ghosh N, Kline J, Roschewski M, LaCasce A, Collins G, Tran T, Lynn J, Chen J, Volkmer J, Agoram B, Huang J, Majeti R, Weissman I, Takimoto C, Chao M, Smith S (2018). CD47 blockade by Hu5F9-G4 and rituximab in non-Hodgkin's lymphoma. N Engl J Med.

[55] Poles W, Nishi E, de Oliveira M, Eugênio A, de Andrade T, Campos A, de Campos R, Vassallo J, Alves A, Scapulatempo Neto C, Paes R, Landman G, Zerbini M, Colleoni G (2019). Targeting the polarization of tumor-associated macrophages and modulating mir-155 expression might be a new approach to treat diffuse large B-cell lymphoma of the elderly. Cancer Immunol Immunother CII.

[56] Moradi-Chaleshtori M, Bandehpour M, Soudi S, Mohammadi-Yeganeh S, Hashemi SM (2021). In vitro and in vivo evaluation of anti-tumoral effect of M1 phenotype induction in macrophages by miR-130 and miR-33 containing exosomes. Cancer Immunol Immunother.

[57] Liu Y, Wei G, Cheng W, Dong Z, Sun H, Lee V, Cha S, Smith D, Kwak L, Qin H (2018). Targeting myeloid-derived suppressor cells for cancer immunotherapy. Cancer Immunol Immunother CII.

[58] Lv M, Wang K, Huang X (2019). Myeloid-derived suppressor cells in hematological malignancies: friends or foes. J Hematol Oncol.

[59] Smyth L, Blunt D, Gatov E, Nagamuthu C, Croxford R, Mozessohn L, Cheung M (2020). Statin and cyclooxygenase-2 inhibitors improve survival in newly diagnosed diffuse large B-cell lymphoma: a large population-based study of 4913 subjects. Br J Haematol.

[60] De Henau O, Rausch M, Winkler D, Campesato L, Liu C, Cymerman D, Budhu S, Ghosh A, Pink M, Tchaicha J, Douglas M, Tibbitts T, Sharma S, Proctor J, Kosmider N, White K, Stern H, Soglia J, Adams J, Palombella V, McGovern K, Kutok J, Wolchok J, Merghoub T (2016). Overcoming resistance to checkpoint blockade therapy by targeting PI3Kγ in myeloid cells. Nature.

[61] Locatelli SL, Careddu G, Serio S, Consonni FM, Maeda A, Viswanadha S, Vakkalanka S, Castagna L, Santoro A, Allavena P, Sica A, Carlo-Stella C (2019). Targeting cancer cells and tumor microenvironment in preclinical and clinical models of hodgkin lymphoma using the dual PI3Kdelta/gamma inhibitor RP6530. Clin Cancer Res.

[62] Xu Z, Ji J, Xu J, Li D, Shi G, Liu F, Ding L, Ren J, Dou H, Wang T, Hou Y (2017). MiR-30a increases MDSC differentiation and immunosuppressive function by targeting SOCS3 in mice with B-cell lymphoma. FEBS J.

[63] Li T, Li X, Zamani A, Wang W, Lee C, Li M, Luo G, Eiler E, Sun H, Ghosh S, Jin J, Murali R, Ruan Q, Shi W, Chen Y (2020). c-Rel Is a myeloid checkpoint for cancer immunotherapy. Nat cancer.

[64] Shaul M, Fridlender Z (2019). Tumour-associated neutrophils in patients with cancer. Nat Rev Clin Oncol.

[65] Ring NG, Herndler-Brandstetter D, Weiskopf K, Shan L, Volkmer JP, George BM, Lietzenmayer M, McKenna KM, Naik TJ, McCarty A, Zheng Y, Ring AM, Flavell RA, Weissman IL (2017). Anti-SIRPalpha antibody immunotherapy enhances neutrophil and macrophage antitumor activity. Proc Natl Acad Sci U S A.

[66] Isles H, Herman K, Robertson A, Loynes C, Prince L, Elks P, Renshaw S (2019). The CXCL12/CXCR4 signaling axis retains neutrophils at inflammatory sites in Zebrafish. Front Immunol.

[67] Wang J, Tannous B, Poznansky M, Chen H (2020). CXCR4 antagonist AMD3100 (plerixafor): from an impurity to a therapeutic agent. Pharmacol Res.

[68] Nie M, Yang L, Bi X, Wang Y, Sun P, Yang H, Liu P, Li Z, Xia Y, Jiang W (2019). Neutrophil extracellular traps induced by IL8 promote diffuse large B-cell lymphoma progression via the TLR9 signaling. Clin Cancer Res.

[69] McWilliams E, Mele J, Cheney C, Timmerman E, Fiazuddin F, Strattan E, Mo X, Byrd J, Muthusamy N, Awan F (2016). Therapeutic CD94/NKG2A blockade improves natural killer cell dysfunction in chronic lymphocytic leukemia. Oncoimmunology.

[70] Kohrt H, Thielens A, Marabelle A, Sagiv-Barfi I, Sola C, Chanuc F, Fuseri N, Bonnafous C, Czerwinski D, Rajapaksa A, Waller E, Ugolini S, Vivier E, Romagné F, Levy R, Bléry M, André P (2014). Anti-KIR antibody enhancement of anti-lymphoma activity of natural killer cells as monotherapy and in combination with anti-CD20 antibodies. Blood.

[71] Lamb M, Rangarajan H, Tullius B, Lee D (2021). Natural killer cell therapy for hematologic malignancies: successes, challenges, and the future. Stem Cell Res Ther.

[72] Zhang C, Hu Y, Shi C (2020). Targeting natural killer cells for tumor immunotherapy. Front Immunol.

[73] Chen X, Song E (2019). Turning foes to friends: targeting cancer-associated fibroblasts. Nat Rev Drug Discov.

[74] Desbois M, Wang Y (2021). Cancer-associated fibroblasts: key players in shaping the tumor immune microenvironment. Immunol Rev.

[75] Biffi G, Tuveson D (2021). Diversity and biology of cancer-associated fibroblasts. Physiol Rev.

[76] Jabłońska-Trypuć A, Matejczyk M, Rosochacki S (2016). Matrix metalloproteinases (MMPs), the main extracellular matrix (ECM) enzymes in collagen degradation, as a target for anticancer drugs. J Enzyme Inhib Med Chem.

[77] Negaard H, Svennevig K, Kolset S, Iversen N, Lothe I, Østenstad B, Sandset P, Iversen P (2009). Alterations in regulators of the extracellular matrix in non-Hodgkin lymphomas. Leuk Lymphoma.

[78] Shen L, Li H, Shi Y, Wang D, Gong J, Xun J, Zhou S, Xiang R, Tan X (2016). M2 tumour-associated macrophages contribute to tumour progression via legumain remodelling the extracellular matrix in diffuse large B cell lymphoma. Sci Rep.

[79] Kesh K, Gupta V, Durden B, Garrido V, Mateo-Victoriano B, Lavania S, Banerjee S (2020). Therapy resistance, cancer stem cells and ECM in cancer: the matrix reloaded. Cancers.

[80] Wang Y, Xiang Y, Xin V, Wang X, Peng X, Liu X, Wang D, Li N, Cheng J, Lyv Y, Cui S, Ma Z, Zhang Q, Xin H (2020). Dendritic cell biology and its role in tumor immunotherapy. J Hematol Oncol.

[81] Grisaru-Tal S, Itan M, Klion A, Munitz A (2020). A new dawn for eosinophils in the tumour microenvironment. Nat Rev Cancer.

[82] Ugel S, Cane S, De Sanctis F, Bronte V (2021). Monocytes in the tumor microenvironment. Annu Rev Pathol.

[83] Vitale I, Manic G, Coussens LM, Kroemer G, Galluzzi L (2019). Macrophages and metabolism in the tumor microenvironment. Cell Metab.

[84] Chiu DK, Tse AP, Xu IM, Di Cui J, Lai RK, Li LL, Koh HY, Tsang FH, Wei LL, Wong CM, Ng IO, Wong CC (2017). Hypoxia inducible factor HIF-1 promotes myeloid-derived suppressor cells accumulation through ENTPD2/CD39L1 in hepatocellular carcinoma. Nat Commun.

[85] Terrén I, Orrantia A, Vitallé J, Zenarruzabeitia O, Borrego F (2019). NK cell metabolism and tumor microenvironment. Front Immunol.

[86] Semenza G (2003). Targeting HIF-1 for cancer therapy. Nat Rev Cancer.

[87] Yu B, Miao ZH, Jiang Y, Li MH, Yang N, Li T, Ding J (2009). c-Jun protects hypoxia-inducible factor-1alpha from degradation via its oxygen-dependent degradation domain in a nontranscriptional manner. Cancer Res.

[88] Zhang H, Chen Z, Miranda RN, Medeiros LJ, McCarty N (2017). Bifurcated BACH2 control coordinates mantle cell lymphoma survival and dispersal during hypoxia. Blood.

[89] Bhalla K, Jaber S, Nahid MN, Underwood K, Beheshti A, Landon A, Bhandary B, Bastian P, Evens AM, Haley J, Polster B, Gartenhaus RB (2018). Role of hypoxia in diffuse large b-cell lymphoma: metabolic repression and selective translation of HK2 facilitates development of DLBCL. Sci Rep.

[90] Minoia C, Quero C, Asselti M, Galise I, Marzano AL, Iacobazzi A, Rana A, Merchionne F, Serrati S, De Tullio G, Quintana G, Casiello M, Maiorano E, Simone G, Zito FA, Iacopino P, Guarini A (2013). Changes in angiogenesis and hypoxia-inducible factor-1alpha protein expression in relapsed/refractory indolent non-Hodgkin lymphomas. Br J Haematol.

[91] Chen W, Hill H, Christie A, Kim M, Holloman E, Pavia-Jimenez A, Homayoun F, Ma Y, Patel N, Yell P, Hao G, Yousuf Q, Joyce A, Pedrosa I, Geiger H, Zhang H, Chang J, Gardner K, Bruick R, Reeves C, Hwang T, Courtney K, Frenkel E, Sun X, Zojwalla N, Wong T, Rizzi J, Wallace E, Josey J, Xie Y (2016). Targeting renal cell carcinoma with a HIF-2 antagonist. Nature.

[92] Choueiri T, Kaelin W (2020). Targeting the HIF2-VEGF axis in renal cell carcinoma. Nat Med.

[93] Courtney K, Ma Y, Diaz de Leon A, Christie A, Xie Z, Woolford L, Singla N, Joyce A, Hill H, Madhuranthakam A, Yuan Q, Xi Y, Zhang Y, Chang J, Fatunde O, Arriaga Y, Frankel A, Kalva S, Zhang S, McKenzie T, Reig Torras O, Figlin R, Rini B, McKay R, Kapur P, Wang T, Pedrosa I, Brugarolas J (2020). HIF-2 complex dissociation, target inhibition, and acquired resistance with PT2385: a first-in-class HIF-2 inhibitor, in patients with clear cell renal cell carcinoma. Clin Cancer Res.

[94] Vinay DS, Ryan EP, Pawelec G, Talib WH, Stagg J, Elkord E, Lichtor T, Decker WK, Whelan RL, Kumara H, Signori E, Honoki K, Georgakilas AG, Amin A, Helferich WG, Boosani CS, Guha G, Ciriolo MR, Chen S, Mohammed SI, Azmi AS, Keith WN, Bilsland A, Bhakta D, Halicka D, Fujii H, Aquilano K, Ashraf SS, Nowsheen S, Yang X (2015). Immune evasion in cancer: mechanistic basis and therapeutic strategies. Semin Cancer Biol.

[95] Zhao R, Song Y, Wang Y, Huang Y, Li Z, Cui Y, Yi M, Xia L, Zhuang W, Wu X, Zhou Y (2019). PD-1/PD-L1 blockade rescue exhausted CD8+ T cells in gastrointestinal stromal tumours via the PI3K/Akt/mTOR signalling pathway. Cell Prolif.

[96] Mimura K, Teh J, Okayama H, Shiraishi K, Kua L, Koh V, Smoot D, Ashktorab H, Oike T, Suzuki Y, Fazreen Z, Asuncion B, Shabbir A, Yong W, So J, Soong R, Kono K (2018). PD-L1 expression is mainly regulated by interferon gamma associated with JAK-STAT pathway in gastric cancer. Cancer Sci.

[97] Yi M, Zhang J, Li A, Niu M, Yan Y, Jiao Y, Luo S, Zhou P, Wu K (2021). The construction, expression, and enhanced anti-tumor activity of YM101: a bispecific antibody simultaneously targeting TGF-β and PD-L1. J Hematol Oncol.

[98] Chu Y, Zhou X, Wang X (2021). Antibody-drug conjugates for the treatment of lymphoma: clinical advances and latest progress. J Hematol Oncol.

[99] Geoerger B, Kang HJ, Yalon-Oren M, Marshall LV, Vezina C, Pappo A, Laetsch TW, Petrilli AS, Ebinger M, Toporski J, Glade-Bender J, Nicholls W, Fox E, DuBois SG, Macy ME, Cohn SL, Pathiraja K, Diede SJ, Ebbinghaus S, Pinto N (2020). Pembrolizumab in paediatric patients with advanced melanoma or a PD-L1-positive, advanced, relapsed, or refractory solid tumour or lymphoma (KEYNOTE-051): interim analysis of an open-label, single-arm, phase 1–2 trial. Lancet Oncol.

[100] Song Y, Wu J, Chen X, Lin T, Cao J, Liu Y, Zhao Y, Jin J, Huang H, Hu J, Luo J, Zhang L, Xue H, Zhang Q, Wang W, Chen C, Feng J, Zhu J (2019). A single-arm, multicenter, phase ii study of camrelizumab in relapsed or refractory classical Hodgkin lymphoma. Clin Cancer Res.

[101] Nie J, Wang C, Liu Y, Yang Q, Mei Q, Dong L, Li X, Liu J, Ku W, Zhang Y, Chen M, An X, Shi L, Brock MV, Bai J, Han W (2019). Addition of low-dose decitabine to anti-PD-1 antibody camrelizumab in relapsed/refractory classical hodgkin lymphoma. J Clin Oncol.

[102] Shi Y, Su H, Song Y, Jiang W, Sun X, Qian W, Zhang W, Gao Y, Jin Z, Zhou J, Jin C, Zou L, Qiu L, Li W, Yang J, Hou M, Zeng S, Zhang Q, Hu J, Zhou H, Xiong Y, Liu P (2019). Safety and activity of sintilimab in patients with relapsed or refractory classical Hodgkin lymphoma (ORIENT-1): a multicentre, single-arm, phase 2 trial. Lancet Haematol.

[103] Song Y, Gao Q, Zhang H, Fan L, Zhou J, Zou D, Li W, Yang H, Liu T, Wang Q, Lv F, Guo H, Yang L, Elstrom R, Huang J, Novotny W, Wei V, Zhu J (2020). Treatment of relapsed or refractory classical Hodgkin lymphoma with the anti-PD-1, tislelizumab: results of a phase 2, single-arm, multicenter study. Leukemia.

[104] Armand P, Rodig S, Melnichenko V, Thieblemont C, Bouabdallah K, Tumyan G, Ozcan M, Portino S, Fogliatto L, Caballero MD, Walewski J, Gulbas Z, Ribrag V, Christian B, Perini GF, Salles G, Svoboda J, Zain J, Patel S, Chen PH, Ligon AH, Ouyang J, Neuberg D, Redd R, Chatterjee A, Balakumaran A, Orlowski R, Shipp M, Zinzani PL (2019). Pembrolizumab in relapsed or refractory primary mediastinal large B-cell lymphoma. J Clin Oncol.

[105] Ding W, LaPlant BR, Call TG, Parikh SA, Leis JF, He R, Shanafelt TD, Sinha S, Le-Rademacher J, Feldman AL, Habermann TM, Witzig TE, Wiseman GA, Lin Y, Asmus E, Nowakowski GS, Conte MJ, Bowen DA, Aitken CN, Van Dyke DL, Greipp PT, Liu X, Wu X, Zhang H, Secreto CR, Tian S, Braggio E, Wellik LE, Micallef I, Viswanatha DS (2017). Pembrolizumab in patients with CLL and Richter transformation or with relapsed CLL. Blood.

[106] Herrera AF, Goy A, Mehta A, Ramchandren R, Pagel JM, Svoboda J, Guan S, Hill JS, Kwei K, Liu EA, Phillips T (2020). Safety and activity of ibrutinib in combination with durvalumab in patients with relapsed or refractory follicular lymphoma or diffuse large B-cell lymphoma. Am J Hematol.

[107] Geoerger B, Zwaan C, Marshall L, Michon J, Bourdeaut F, Casanova M, Corradini N, Rossato G, Farid-Kapadia M, Shemesh C, Hutchinson K, Donaldson F, Liao M, Caron H, Trippett T (2020). Atezolizumab for children and young adults with previously treated solid tumours, non-Hodgkin lymphoma, and Hodgkin lymphoma (iMATRIX): a multicentre phase 1–2 study. Lancet Oncol.

[108] Pascual M, Mena-Varas M, Robles EF, Garcia-Barchino MJ, Panizo C, Hervas-Stubbs S, Alignani D, Sagardoy A, Martinez-Ferrandis JI, Bunting KL, Meier S, Sagaert X, Bagnara D, Guruceaga E, Blanco O, Celay J, Martinez-Baztan A, Casares N, Lasarte JJ, MacCarthy T, Melnick A, Martinez-Climent JA, Roa S (2019). PD-1/PD-L1 immune checkpoint and p53 loss facilitate tumor progression in activated B-cell diffuse large B-cell lymphomas. Blood.

[109] Wu X, Gu Z, Chen Y, Chen B, Chen W, Weng L, Liu X (2019). Application of PD-1 blockade in cancer immunotherapy. Comput Struct Biotechnol J.

[110] Havel J, Chowell D, Chan T (2019). The evolving landscape of biomarkers for checkpoint inhibitor immunotherapy. Nat Rev Cancer.

[111] Mitsuiki N, Schwab C, Grimbacher B (2019). What did we learn from CTLA-4 insufficiency on the human immune system?. Immunol Rev.

[112] Rowshanravan B, Halliday N, Sansom DM (2018). CTLA-4: a moving target in immunotherapy. Blood.

[113] Tang F, Du X, Liu M, Zheng P, Liu Y (2018). Anti-CTLA-4 antibodies in cancer immunotherapy: selective depletion of intratumoral regulatory T cells or checkpoint blockade?. Cell Biosci.

[114] Sharma A, Subudhi SK, Blando J, Scutti J, Vence L, Wargo J, Allison JP, Ribas A, Sharma P (2019). Anti-CTLA-4 immunotherapy does not deplete FOXP3(+) regulatory T cells (Tregs) in human cancers. Clin Cancer Res.

[115] Ingram J, Blomberg O, Rashidian M, Ali L, Garforth S, Fedorov E, Fedorov A, Bonanno J, Le Gall C, Crowley S, Espinosa C, Biary T, Keliher E, Weissleder R, Almo S, Dougan S, Ploegh H, Dougan M (2018). Anti-CTLA-4 therapy requires an Fc domain for efficacy. Proc Natl Acad Sci USA.

[116] Larkin J (2015). Combined nivolumab and ipilimumab or monotherapy in untreated melanoma. N Engl J Med.

[117] Armand P, Lesokhin A, Borrello I, Timmerman J, Gutierrez M, Zhu L, Popa McKiver M, Ansell SM (2021). A phase 1b study of dual PD-1 and CTLA-4 or KIR blockade in patients with relapsed/refractory lymphoid malignancies. Leukemia.

[118] Patel S, Weirather J, Lipschitz M, Lako A, Chen P, Griffin G, Armand P, Shipp M, Rodig S (2019). The microenvironmental niche in classic Hodgkin lymphoma is enriched for CTLA-4-positive T cells that are PD-1-negative. Blood.

[119] Tuscano JM, Maverakis E, Groshen S, Tsao-Wei D, Luxardi G, Merleev AA, Beaven A, DiPersio JF, Popplewell L, Chen R, Kirschbaum M, Schroeder MA, Newman EM (2019). A phase I study of the combination of rituximab and ipilimumab in patients with relapsed/refractory B-cell lymphoma. Clin Cancer Res.

[120] Jayaraman J, Mellody M, Hou A, Desai R, Fung A, Pham A, Chen Y, Zhao W (2020). CAR-T design: elements and their synergistic function. EBioMedicine.

[121] Srivastava S, Riddell SR (2015). Engineering CAR-T cells: design concepts. Trends Immunol.

[122] Schuster S, Bishop M, Tam C, Waller E, Borchmann P, McGuirk J, Jäger U, Jaglowski S, Andreadis C, Westin J, Fleury I, Bachanova V, Foley S, Ho P, Mielke S, Magenau J, Holte H, Pantano S, Pacaud L, Awasthi R, Chu J, Anak Ö, Salles G, Maziarz R (2019). Tisagenlecleucel in adult relapsed or refractory diffuse large B-cell lymphoma. N Engl J Med.

[123] Locke FL, Ghobadi A, Jacobson CA, Miklos DB, Lekakis LJ, Oluwole OO, Lin Y, Braunschweig I, Hill BT, Timmerman JM, Deol A, Reagan PM, Stiff P, Flinn IW, Farooq U, Goy A, McSweeney PA, Munoz J, Siddiqi T, Chavez JC, Herrera AF, Bartlett NL, Wiezorek JS, Navale L, Xue A, Jiang Y, Bot A, Rossi JM, Kim JJ, Go WY (2019). Long-term safety and activity of axicabtagene ciloleucel in refractory large B-cell lymphoma (ZUMA-1): a single-arm, multicentre, phase 1–2 trial. Lancet Oncol.

[124] Jacobson CA, Chavez JC, Sehgal AR, William BM, Munoz J, Salles GA, Casulo C, Munshi PN, Maloney DG, De Vos S, Reshef R, Leslie LA, Yakoub-Agha I, Oluwole OO, Fung HC, Plaks V, Yang Y, Lee J, Avanzi MP, Neelapu SS (2020). Interim analysis of ZUMA-5: A phase II study of axicabtagene ciloleucel (axi-cel) in patients (pts) with relapsed/refractory indolent non-Hodgkin lymphoma (R/R iNHL). J Clin Oncol.

[125] Wang M, Munoz J, Goy A, Locke FL, Jacobson CA, Hill BT, Timmerman JM, Holmes H, Jaglowski S, Flinn IW, McSweeney PA, Miklos DB, Pagel JM, Kersten M-J, Milpied N, Fung H, Topp MS, Houot R, Beitinjaneh A, Peng W, Zheng L, Rossi JM, Jain RK, Rao AV, Reagan PM (2020). KTE-X19 CAR T-cell therapy in relapsed or refractory mantle-cell lymphoma. N Engl J Med.

[126] Abramson JS, Palomba ML, Gordon LI, Lunning MA, Wang M, Arnason J, Mehta A, Purev E, Maloney DG, Andreadis C, Sehgal A, Solomon SR, Ghosh N, Albertson TM, Garcia J, Kostic A, Mallaney M, Ogasawara K, Newhall K, Kim Y, Li D, Siddiqi T (2020). Lisocabtagene maraleucel for patients with relapsed or refractory large B-cell lymphomas (TRANSCEND NHL 001): a multicentre seamless design study. Lancet.

[127] Brudno J, Lam N, Vanasse D, Shen Y, Rose J, Rossi J, Xue A, Bot A, Scholler N, Mikkilineni L, Roschewski M, Dean R, Cachau R, Youkharibache P, Patel R, Hansen B, Stroncek D, Rosenberg S, Gress R, Kochenderfer J (2020). Safety and feasibility of anti-CD19 CAR T cells with fully human binding domains in patients with B-cell lymphoma. Nat Med.

[128] Ramos C, Grover N, Beaven A, Lulla P, Wu M, Ivanova A, Wang T, Shea T, Rooney C, Dittus C, Park S, Gee A, Eldridge P, McKay K, Mehta B, Cheng C, Buchanan F, Grilley B, Morrison K, Brenner M, Serody J, Dotti G, Heslop H, Savoldo B (2020). Anti-CD30 CAR-T cell therapy in relapsed and refractory hodgkin lymphoma. J Clin Oncol.

[129] Fry T, Shah N, Orentas R, Stetler-Stevenson M, Yuan C, Ramakrishna S, Wolters P, Martin S, Delbrook C, Yates B, Shalabi H, Fountaine T, Shern J, Majzner R, Stroncek D, Sabatino M, Feng Y, Dimitrov D, Zhang L, Nguyen S, Qin H, Dropulic B, Lee D, Mackall C (2018). CD22-targeted CAR T cells induce remission in B-ALL that is naive or resistant to CD19-targeted CAR immunotherapy. Nat Med.

[130] Lynn RC, Weber EW, Sotillo E, Gennert D, Xu P, Good Z, Anbunathan H, Lattin J, Jones R, Tieu V, Nagaraja S, Granja J, de Bourcy CFA, Majzner R, Satpathy AT, Quake SR, Monje M, Chang HY, Mackall CL (2019). c-Jun overexpression in CAR T cells induces exhaustion resistance. Nature.

[131] Huang R, Li X, He Y, Zhu W, Gao L, Liu Y, Gao L, Wen Q, Zhong J, Zhang C, Zhang X (2020). Recent advances in CAR-T cell engineering. J Hematol Oncol.

[132] Amor C, Feucht J, Leibold J, Ho Y, Zhu C, Alonso-Curbelo D, Mansilla-Soto J, Boyer J, Li X, Giavridis T, Kulick A, Houlihan S, Peerschke E, Friedman S, Ponomarev V, Piersigilli A, Sadelain M, Lowe S (2020). Senolytic CAR T cells reverse senescence-associated pathologies. Nature.

[133] Dana H, Chalbatani G, Jalali S, Mirzaei H, Grupp S, Suarez E, Rapôso C, Webster T (2021). CAR-T cells: early successes in blood cancer and challenges in solid tumors. Acta Pharmaceutica Sinica B.

[134] Henze J, Tacke F, Hardt O, Alves F, Al Rawashdeh W (2020). Enhancing the efficacy of CAR T cells in the tumor microenvironment of pancreatic cancer. Cancers.

[135] Rodriguez-Garcia A, Lynn R, Poussin M, Eiva M, Shaw L, O'Connor R, Minutolo N, Casado-Medrano V, Lopez G, Matsuyama T, Powell D (2021). CAR-T cell-mediated depletion of immunosuppressive tumor-associated macrophages promotes endogenous antitumor immunity and augments adoptive immunotherapy. Nat Commun.

[136] Liu G, Rui W, Zhao X, Lin X (2021). Enhancing CAR-T cell efficacy in solid tumors by targeting the tumor microenvironment. Cell Mol Immunol.

[137] Larson R, Maus M (2021). Recent advances and discoveries in the mechanisms and functions of CAR T cells. Nat Rev Cancer.

[138] Klichinsky M, Ruella M, Shestova O, Lu XM, Best A, Zeeman M, Schmierer M, Gabrusiewicz K, Anderson NR, Petty NE, Cummins KD, Shen F, Shan X, Veliz K, Blouch K, Yashiro-Ohtani Y, Kenderian SS, Kim MY, O'Connor RS, Wallace SR, Kozlowski MS, Marchione DM, Shestov M, Garcia BA, June CH, Gill S (2020). Human chimeric antigen receptor macrophages for cancer immunotherapy. Nat Biotechnol.

[139] Liu E, Marin D, Banerjee P, Macapinlac HA, Thompson P, Basar R, Nassif Kerbauy L, Overman B, Thall P, Kaplan M, Nandivada V, Kaur I, Nunez Cortes A, Cao K, Daher M, Hosing C, Cohen EN, Kebriaei P, Mehta R, Neelapu S, Nieto Y, Wang M, Wierda W, Keating M, Champlin R, Shpall EJ, Rezvani K (2020). Use of CAR-transduced natural killer cells in CD19-positive lymphoid tumors. N Engl J Med.

[140] Zhang L, Tian L, Dai X, Yu H, Wang J, Lei A, Zhu M, Xu J, Zhao W, Zhu Y, Sun Z, Zhang H, Hu Y, Wang Y, Xu Y, Church G, Huang H, Weng Q, Zhang J (2020). Pluripotent stem cell-derived CAR-macrophage cells with antigen-dependent anti-cancer cell functions. J Hematol Oncol.

[141] Sharma P, Hu-Lieskovan S, Wargo J, Ribas A (2017). Primary, adaptive, and acquired resistance to cancer immunotherapy. Cell.

[142] Chen D, Mellman I (2017). Elements of cancer immunity and the cancer-immune set point. Nature.

[143] Goliwas KF, Deshane JS, Elmets CA, Athar M (2021). Moving immune therapy forward targeting TME. Physiol Rev.

[144] Rotte A (2019). Combination of CTLA-4 and PD-1 blockers for treatment of cancer. J Exp Clin Cancer Res CR.

[145] Scott E, Gocher A, Workman C, Vignali D (2021). Regulatory T cells. Barriers of immune infiltration into the tumor microenvironment. Front Immunol.

[146] Galon J, Bruni D (2019). Approaches to treat immune hot, altered and cold tumours with combination immunotherapies. Nat Rev Drug Discov.

[147] Scott D, Gascoyne R (2014). The tumour microenvironment in B cell lymphomas. Nat Rev Cancer.

[148] Cencini E, Fabbri A, Schiattone L, Sicuranza A, Mecacci B, Granai M, Mancini V, Lazzi S, Bocchia M, Leoncini L (2020). Prognostic impact of tumor-associated macrophages, lymphocyte-to-monocyte and neutrophil-to-lymphocyte ratio in diffuse large B-cell lymphoma. Am J Blood Res.

[149] Keane C, Law SC, Gould C, Birch S, Sabdia MB, Merida de Long L, Thillaiyampalam G, Abro E, Tobin JW, Tan X, Xu-Monette ZY, Young KH, Gifford G, Gabreilli S, Stevenson WS, Gill A, Talaulikar D, Jain S, Hernandez A, Halliday SJ, Bird R, Cross D, Hertzberg M, Gandhi MK (2020). LAG3: a novel immune checkpoint expressed by multiple lymphocyte subsets in diffuse large B-cell lymphoma. Blood Adv.

[150] Schwaller J, Schneider P, Mhawech-Fauceglia P, McKee T, Myit S, Matthes T, Tschopp J, Donze O, Le Gal FA, Huard B (2007). Neutrophil-derived APRIL concentrated in tumor lesions by proteoglycans correlates with human B-cell lymphoma aggressiveness. Blood.

[151] Jia Q, Qin D, He F, Xie Q, Ying Z, Zhang Y, Song Y, Cheng J, Zuo X, Xu L, Fang H, Hu C, Peng L, Jin T, Shi Z, Alexander P, Wang Y, Liu Y, Han W, Zhu J, Wang P, Li Q, Zhu B (2021). Peripheral eosinophil counts predict efficacy of anti-CD19 CAR-T cell therapy against B-lineage non-Hodgkin lymphoma. Theranostics.

[152] Kiyasu J, Miyoshi H, Hirata A, Arakawa F, Ichikawa A, Niino D, Sugita Y, Yufu Y, Choi I, Abe Y, Uike N, Nagafuji K, Okamura T, Akashi K, Takayanagi R, Shiratsuchi M, Ohshima K (2015). Expression of programmed cell death ligand 1 is associated with poor overall survival in patients with diffuse large B-cell lymphoma. Blood.

[153] Qiu L, Zheng H, Zhao X (2019). The prognostic and clinicopathological significance of PD-L1 expression in patients with diffuse large B-cell lymphoma: a meta-analysis. BMC Cancer.

[154] Kwon D, Kim S, Kim PJ, Go H, Nam SJ, Paik JH, Kim YA, Kim TM, Heo DS, Kim CW, Jeon YK (2016). Clinicopathological analysis of programmed cell death 1 and programmed cell death ligand 1 expression in the tumour microenvironments of diffuse large B cell lymphomas. Histopathology.

[155] Ishikawa E, Nakamura M, Shimada K, Tanaka T, Satou A, Kohno K, Sakakibara A, Furukawa K, Yamamura T, Miyahara R, Nakamura S, Kato S, Fujishiro M (2020). Prognostic impact of PD-L1 expression in primary gastric and intestinal diffuse large B-cell lymphoma. J Gastroenterol.

[156] Pollari M, Brück O, Pellinen T, Vähämurto P, Karjalainen-Lindsberg M, Mannisto S, Kallioniemi O, Kellokumpu-Lehtinen P, Mustjoki S, Leivonen S, Leppä S (2018). PD-L1 tumor-associated macrophages and PD-1 tumor-infiltrating lymphocytes predict survival in primary testicular lymphoma. Haematologica.

[157] Lim B, Lin Y, Navin N (2020). Advancing cancer research and medicine with single-cell genomics. Cancer Cell.

[158] Moncada R, Barkley D, Wagner F, Chiodin M, Devlin JC, Baron M, Hajdu CH, Simeone DM, Yanai I (2020). Integrating microarray-based spatial transcriptomics and single-cell RNA-seq reveals tissue architecture in pancreatic ductal adenocarcinomas. Nat Biotechnol.

[159] Zhao T, Lyu S, Lu G, Juan L, Zeng X, Wei Z, Hao J, Peng J (2021). SC2disease: a manually curated database of single-cell transcriptome for human diseases. Nucleic Acids Res.

[160] Yuan H, Yan M, Zhang G, Liu W, Deng C, Liao G, Xu L, Luo T, Yan H, Long Z, Shi A, Zhao T, Xiao Y, Li X (2019). CancerSEA: a cancer single-cell state atlas. Nucleic Acids Res.

[161] He L, Vanlandewijck M, Mae MA, Andrae J, Ando K, Del Gaudio F, Nahar K, Lebouvier T, Lavina B, Gouveia L, Sun Y, Raschperger E, Segerstolpe A, Liu J, Gustafsson S, Rasanen M, Zarb Y, Mochizuki N, Keller A, Lendahl U, Betsholtz C (2018). Single-cell RNA sequencing of mouse brain and lung vascular and vessel-associated cell types. Sci Data.

[162] Franzen O, Gan LM, Bjorkegren JLM (2019). PanglaoDB: a web server for exploration of mouse and human single-cell RNA sequencing data. Database (Oxford).

[163] Zhang X, Lan Y, Xu J, Quan F, Zhao E, Deng C, Luo T, Xu L, Liao G, Yan M, Ping Y, Li F, Shi A, Bai J, Zhao T, Li X, Xiao Y (2019). Cell Marker: a manually curated resource of cell markers in human and mouse. Nucleic Acids Res.

[164] Cao ZJ, Wei L, Lu S, Yang DC, Gao G (2020). Searching large-scale scRNA-seq databases via unbiased cell embedding with Cell BLAST. Nat Commun.

[165] Björkegren JLM, Franzén O, Gorodkin J (2020). alona: a web server for single-cell RNA-seq analysis. Bioinformatics.

[166] Gong B, Wang R, Xu H, Miao M, Yao Z (2019). Nanotherapy targeting the tumor microenvironment. Curr Cancer Drug Targets.

[167] Yang M, Li J, Gu P, Fan X (2021). The application of nanoparticles in cancer immunotherapy: targeting tumor microenvironment. Bioact Mater.

[168] Cheng Z, Li M, Dey R, Chen Y (2021). Nanomaterials for cancer therapy: current progress and perspectives. J Hematol Oncol.

[169] Del Piccolo N, Shirure V, Bi Y, Peter Goedegebuure S, Gholami S, Hughes C, Fields R, George S (2021). Tumor-on-chip modeling of organ-specific cancer and metastasis. Adv Drug Deliv Rev.

[170] Garcia-Carbonero R, Martin MG, Gallego RA, Mercade TM, Martinez MCR, Guillen-Ponce C, Vidal N, Real FX, Moreno R, Maliandi V, Mato-Berciano A, Bazan-Peregrino M, Capella G, Alemany R, Blasi E, Blasco C, Cascallo M, Salazar R (2019). Systemic administration of the hyaluronidase-expressing oncolytic adenovirus VCN-01 in patients with advanced or metastatic pancreatic cancer: first-in-human clinical trial. Ann Oncol.

